# Supplementary material for: HIV testing, care and viral suppression among men who have sex with men and transgender individuals in Johannesburg, South Africa
Source: PLoS One. 2020 Jun 17;15(6):e0234384. doi: 10.1371/journal.pone.0234384 (PMC7299351; doi:10.1371/journal.pone.0234384)
Supplement: S1 Data — (DOCX) [file pone.0234384.s001.docx]

# Appendix 1: Respondent Driven Sampling recruitment diagnostics

In line with guidance^1^ we conducted monitoring of the RDS recruitment every two weeks and made recommendations to the data collection team on the basis of findings. We examined RDS recruitment trees, return of coupons, assessed sample composition by sociodemographic characteristics, and examined convergence on key outcomes overall and by seed (assessed for potential ‘bottlenecks’ in which recruitment gets ‘stuck’ within sub-groups).

## **Findings**

### **Waves and seeds**

Nine seed participants recruited a total of 301 participants, though 295/301, 98% of all participants, came from 5 seeds of between 6 and 23 waves, Table A1. The largest recruitment chain of Seed 2 recruited 120/301 (39.9%) of participants, Table A2.

Participants were given 2 coupons each, going down to one coupon each in August 2017 in an effort to slow recruitment in existing chains and start chains with new seeds from under-represented population groups.

**Table A1: Number of participants recruited by wave**

| Wave | 0 | 1 | 2 | 3 | 4 | 5 | 6 | 7 | 8 | 9 | 10 | 11 | 12 |
| --- | --- | --- | --- | --- | --- | --- | --- | --- | --- | --- | --- | --- | --- |
| No. Participants | 9 | 11 | 12 | 18 | 29 | 29 | 37 | 29 | 26 | 27 | 26 | 17 | 7 |

| Wave | 13 | 14 | 15 | 16 | 17 | 18 | 19 | 20 | 21 | 22 | 23 |
| --- | --- | --- | --- | --- | --- | --- | --- | --- | --- | --- | --- |
| No. Participants | 6 | 4 | 3 | 2 | 2 | 2 | 1 | 1 | 1 | 1 | 1 |

**Table A2: Number of participants and recruitment waves recruited via each seed**

| Seed | Number of participants (%) | Waves |
| --- | --- | --- |
| 1 | 52 (17.3%) | 18 |
| 2 | 120 (39.9%) | 23 |
| 3 | 37 (12.3%) | 10 |
| 4 | 1 (0.3%) | 0 |
| 5 | 54 (17.9%) | 13 |
| 6 | 2 (0.6%) | 1 |
| 7 | 1 (0.3%) | 0 |
| 8 | 2 (0.6%) | 1 |
| 9 | 32 (10.6%) | 6 |

### **Coupon receipt**

The majority of participants received a coupon from a close friend or friend and only 9 (3.3%) of participants reported that they received their coupon from a stranger, which violates the RDS assumption that participants receive a coupon from someone within their social network (and the recruitment instructions). Consistent with whom they received a coupon from, the majority of participants reported that they received it from or near their home. There were 22 participants (7.4%) who reported receiving their coupon outside the study clinic which could imply distributions to individuals who just happened to be passing rather than distribution to an individual’s social network, but this is not certain.

A minority of 52/301 (17.3%) of participants reported that they had received more than one coupon distribution attempt, which suggests that recruitment did not get stuck within a small group trying to recruit each other.

**Table A3: Characteristics of coupon receipt reported by participants**

|  | **n** | **%** |
| --- | --- | --- |
| **Relationship to person from whom received coupon** |  |  |
| Close friend | 136 | 49.3 |
| Friend | 113 | 40.9 |
| Acquaintance | 14 | 5.1 |
| Stranger | 9 | 3.3 |
| Other | 4 | 1.5 |
|  |  |  |
| **Where received coupon** |  |  |
| At/near home | 199 | 67.2 |
| At/near work | 16 | 5.4 |
| On street | 29 | 9.8 |
| Bar/club | 8 | 2.7 |
| Outside the study | 22 | 7.4 |
| At school | 6 | 2.0 |
| Other | 16 | 5.4 |
|  |  |  |
| **Apart from the person who gave you the coupon you brought today has anyone else tried to give you a coupon?** |  |  |
| no | 248 | 82.7 |
| yes | 52 | 17.3 |
| **How many times?** |  |  |
| 1 | 30 | 60.0 |
| 2 | 10 | 20.0 |
| 3 | 6 | 12.0 |
| 4 | 2 | 4.0 |
| 5 | 0 | 0.0 |
| 6 | 0 | 0.0 |
| 7 | 1 | 2.0 |
| 8 | 1 | 2.0 |

**Figure A1: Recruitment tree by neighbourhood**

Neighbourhoods are named when listed by at least 10 participants and otherwise are included in ‘Other’.

Aiming to capture the diversity of the MSM/TG population in Johannesburg, we attempted to initiate recruitment chains from seeds of different population group, age, sexual and gender identities. However, we were unable to recruit more than one White participant beyond the seeds, and no Asian participants were recruited, (Table 1, main paper).

There were 158/301 participants from Soweto (52.5% unweighted, 55.3%, 95% CI 45.7-65% RDS-weighted), though 28.7% of the population in Johannesburg as a whole was from Soweto, as of the 2011 census^2^. There is some suggestion of clustering in recruitment by neighbourhood of residence (Figure A1), though all longer recruitment chains (>Wave 1) included participants from different neighbourhoods.

### **Convergence of Estimates**

Each convergence plot shows the cumulative RDS-II weighted proportion of the population estimate as the sample recruitment progressed. We examined key sociodemographic characteristics, HIV testing, prevalence and viral suppression.

**Figure A2a. Age: proportion under 25 years**

**Figure A2b. Educational attainment**

**Figure A2c. Employment**

**Figure A2d. Income in the last month: proportion in each category**

**Figure A2e. Sexual identity**

**Figure A2f. Gender identity**

**Figure A2g. HIV: proportion positive**

**Figure A2h. Tested for HIV in the previous 6 months amongst HIV-negative MSM/TG**

**Figure A2i. Viral suppression amongst HIV-positive MSM/TG**

Convergence by age (proportion of the sample under age 25 years) was reasonable, as was convergence in estimates of gender identity. There was some evidence that the proportion bisexual individuals began to rise and then fall again towards the end of sample recruitment. The proportion of unemployed individuals might also have continued to rise had recruitment continued.

The convergence of the HIV estimate was reasonable, as was the proportion of those HIV-positive MSM/TG who were virally suppressed below 50 copies/ml plasma.

### **Bottlenecks**

We show the bottleneck plot for age only to protect the privacy of seed participants, of whom there are fewer than ten.

**Figure A3. Age: proportion under 25 years**

One recruitment chain, the longest, tended to have a lower proportion of those aged under 25 years (Figure A3), where overall this estimate had appeared to be converging across the chains (Figure A2a).

The cumulative weighted estimated proportion of those identifying as gay or homosexual did not appear to vary greatly by seed, nor that as recruitment progressed across different chains, that there was a general trend towards higher proportions identifying as one sexual identity or another. This was also true of gender identity.

There was one recruitment chain, the longest and the youngest, that tended to have a lower cumulative weighted proportion testing HIV-positive.

### **Discussion**

The majority of participants reported that they knew the person who had given them a coupon, a key assumption of the RDS method (mutuality). With the exception of the 4 seeds that were unproductive or recruited only one further participant, a good number of sample waves was achieved. The majority of primary outcome estimates (HIV status, viral suppression amongst those HIV-positive and HIV testing amongst those HIV-negative) appeared to converge reasonably by the end of sample recruitment.

The Johannesburg 2011 census found that 77.1% of the population of Johannesburg was Black African, 5.6% Coloured, 4.9% Indian and 12.4% White. The percentage of MSM/TG identifying themselves as Coloured in our study was 2.8%, 95% CI 0-5.9%, 0.6% identified as White, 95% CI 0-1.5%, 1.2% as Other 95% 0-4.8%, and 94.8% as Black Africa, 95% CI 89.2-100.0%)^3^. We would expect that the MSM/TG proportion of the population would not vary greatly by population group, so this suggests that we under-recruited White and Asian participants. This could have been due to a tendency for RDS studies to under-recruit amongst the highest status population groups in a society (we had a high proportion of unemployed participants), that White or Asian participants given coupons disproportionately did not wish to take part or were not given coupons in the first place, suggesting underlying socialising patterns amongst MSM/TG in Johannesburg.

Like many RDS surveys of MSM in sub-Saharan Africa, the mean age was quite young^4^. This might reflect age and cohort effects in MSM socialising patterns and difficulties in reaching older MSM/TG are reported by community based groups in many parts of the region^5^. Additionally, there was a higher proportion of our sample reporting that they identified as gay/homosexual and a lower proportion identifying as heterosexual than was found in a 2008 study of MSM in Soweto^6^. It is possible that our survey, like many RDS surveys among MSMN in SSA, reached fewer who are perhaps less likely to be well connected to social networks of gay MSM.

A large proportion of participants came from one recruitment chain of 23 waves. This chain tended to be younger, less likely to identify as gay and less likely to be HIV-positive than other chains, which might be a reflection of penetrating deeper into social networks. There was not a uniform trend towards younger, HIV-negative, non-gay identified MSM/TG in the other recruitment chains though.

Convergence of our primary outcomes and of other key sociodemographic characteristics including sexual identity and gender identity was reasonable. One recruitment chain tended to be somewhat younger and somewhat less likely to be HIV-positive. As younger MSM/TG were less likely to be HIV-positive overall, it is unclear what characteristics might be driving any underlying socialising patterns.

## **Supplementary References**

1. Gile KJ, Johnston LG, Salganik MJ. Diagnostics for Respondent-driven Sampling. *J R Stat Soc Ser A Stat Soc* 2015; **178**(1): 241-69.

2. Census 2011. 2012. <http://www.statssa.gov.za/?page_id=993&id=city-of-johannesburg-municipality> (accessed 21 February 2019 2019).

3. Census 2011 Municipal Report: Gauteng: Statistics South Africa, 2012.

4. Johnson LF, Mulongeni P, Marr A, Lane T. Age bias in survey sampling and implications for estimating HIV prevalence in men who have sex with men: insights from mathematical modelling. *Epidemiol Infect* 2018; **146**(8): 1036-42.

5. Bourne A, Fearon E, Nutland W. Mapping and appraisal of HIV prevention and care interventions among men who have sex wioth men in Kenya, Uganda, Tanzania and Zimbabwe: a report of the SHARP programme: International HIV/AIDS Alliance

Sigma Research, 2016.

6. Lane T, Raymond HF, Dladla S, et al. High HIV prevalence among men who have sex with men in Soweto, South Africa: results from the Soweto Men's Study. *AIDS Behav* 2011; **15**(3): 626-34.
