# Supplementary material for: HIV testing, care and viral suppression among men who have sex with men and transgender individuals in Johannesburg, South Africa
Source: PLoS One. 2020 Jun 17;15(6):e0234384. doi: 10.1371/journal.pone.0234384 (PMC7299351; doi:10.1371/journal.pone.0234384)
Supplement: S2 Data — (DOCX) [file pone.0234384.s002.docx]

# Appendix 2: Viral Suppression according to different viral load cut-offs

Table A4 shows the proportion of HIV-positive MSM/TG who were virally suppressed according to different viral load cut-offs. The intention is to aid comparability across contexts and over time. We have chosen <50 viral copies/ml plasma as our primary definition of suppression, in line with 2019 South African Clinical guidelines[1], but we also report <200 copies/ml in line with CDC guidelines[2], <400 copies/ml in line with the South African treatment guidelines at the time our data was collected in 2017[3], and <1000 copies/ml.

**Table A4: Viral suppression amongst HIV-positive MSM/TG in Johannesburg, n=118**

**Viral load cut-off Virally suppressed of all HIV-Positive, n=118 Virally suppressed of those reporting current ART, n=39**

**Indicating suppression**

**n RDS % n RDS %**

<200 copies/ml 64 49.4 32 84.6

<400 copies/ml 68 53.9 33 87.4

<1000 copies/ml 74 60.1 36 94.4

1. 2019 ART Clinical Guidelines for the Management of HIV in Adults, Pregnancy, Adolescents, Children, Infants and Neonates Pretoria, South Africa: Republic of South Africa National Department of Health, 2019 October 2019. Report No.

2. Centers for Disease Control and Prevention. Questions and Answers for the General Public: Revised Recommendations for HIV Testing of Adults, Adolescents, and Pregnant Women in Health Care Settings. Accessed 3 September 2017, <http://www.cdc.gov/hiv/topics/testing/resources/qa/qa_general-public.htm>.

3. National consolidated guidelines for the prevention of mother-to-child transmission of HIV (PMTCT) and the management of HIV in children, adolescents and adults Pretoria: Republic of South Africa National Department of Health; 2015.
