# Supplementary material for: HIV testing, care and viral suppression among men who have sex with men and transgender individuals in Johannesburg, South Africa
Source: PLoS One. 2020 Jun 17;15(6):e0234384. doi: 10.1371/journal.pone.0234384 (PMC7299351; doi:10.1371/journal.pone.0234384)
Supplement: S3 Data — (PDF) [file pone.0234384.s003.pdf]

TRANSFORM Survey Instrument v.4

Date today: [year/months/day]

Site name: _______________

Study Staff name: _______________

Participant ID number: _______________

Thank you for agreeing to complete this survey. We will now ask you a few questions about yourself. Please remember that you do not have to answer any questions you do not want to answer and you can stop the survey at any time.

PRACTICE QUESTIONS

There are three main types of questions .. the most common has small ROUND buttons for the answers and you can click only one answer. Once you have clicked on an answer the question will disappear and the next one will be shown. If you have made a mistake, just click the left-facing arrow head ("BACK") button on the bottom left hand side of the screen. Try this one ...

How handsome are you?

Handsome
Very handsome
Incredibly handsome

Other questions have small SQUARE buttons for the answers and you can click as many of the answers as you like. Once you have clicked on an answer the question will not dissappear - when you have finished you need to click the right-facing arrow head ("NEXT") button on the bottom right hand side. If you have made a mistake, just click the left-facing arrow head ("BACK") button on the bottom left hand side. Try this one ...

Which of the following sports do you watch on television?
Notice these questions often have an "OTHER" option - if you click this you need to type in your other answer. Click "OTHER" and write in "Boxing" (or any other sport you watch).

Soccer
Rugby
Cricket
Other – please say which: (type in)

A few questions ask you when you last did something and offer you a calender to pick the exact day, month and year. When this happens you can navigate the months and years using the left and right arrows on the calendar. When you have picked you date for your answer, click "Set" to record it. Once you have clicked on "Set" the question will disappear and the next one will be shown. Try this one ...

When did you last speak to one of your family members?

Select date (day, month, year)

AX. Coupon management

We are now going to start the survey properly - please answer every question honestly. Please remember that you do not have to answer any questions you do not want to answer and you can stop the survey at any time. All your responses are totally confidential.

The first set of questions are about how you found out about the study, and where you got the coupon.

AX1. How do you know the person who gave you the coupon to participate in the survey?
Close friend
Friend
Acquaintance
Stranger

Other

Q1a, If Q1 = Other ask, How else do you know them?

[write in]

AX2. Where were you when you received a coupon to participate in the study?
At or near home
At or near work
Out and about on the street
At a bar/club
Outside this office
Elsewhere (say where ____________)

AX3. If you had participated in the survey first, do you think that you might have given a coupon to the person who gave you one?
Yes
No

AX4. Apart from the person who gave you the coupon you brought today, has anyone else offered you a coupon?
Yes
No

AX5. If yes, how many times has this happened?

[____] times

A. QUESTIONS ABOUT YOU (1)

The first set of questions are about you .. your age, where you live, and where you were born, for example. The survey has started - all your answers are confidential. Please answer questions truthfully.

A1. How old are you?

_____ (years)

A2. Which neighbourhood do you live in?

[SHOW IF SITENAME = NAIROBI]

Dagoretti
Embakasi
Kamukunji
Kasarani
Langata
Makadara
Starehe
Westlands
Other (please specify) ____________

[SHOW IF SITENAME = Johannesburg SOUTH AFRICA]

Braamfontein
Diepkloof,
Hillbrow
Orange Farm
Rosebank
Rosetonville
Sandton,
Soweto
Yeoville
Other (please specify) ____________

A3. Were you born in [Kenya / South Africa]?

No
Yes **[jump to A5]**

A4. [If A3 is no] Were you born in another country in Africa?

Yes
No

A4a. [If A4 is yes] Which country in Africa were you born?

[Select from list of countries]

A5. Were you born in [SITE NAME] ?

No

Yes

A6. [If A5 is no] Which town were you born in?

Town/Village ____________

A7. How many years have you been living around [SITE NAME]?

[Type in]

A. QUESTIONS ABOUT YOU (2)

A8. What is your highest level of education completed?

[SHOW IF SOUTH AFRICA]

No education
Primary school
Junior high school
High school or technical secondary school
College, university or higher education

[SHOW IF KENYA]

No education
Primary school
Secondary school
College, university or higher education

A9. What best describes your current employment status?

Employed full-time
Employed part-time
Self-employed
Unemployed or between jobs
Other (say what_____________)

A11. What was your income last month?

[SHOW IF KENYA]

KSH [enter number]

[SHOW IF SOUTH AFRICA]

ZAR [enter number]

A12. Including yourself, how many people depend on this income?

[enter number]

A. QUESTIONS ABOUT YOU (3)

A13. What is your religious affiliation?

Christianity
Islam
Hinduism
Other
None

A14. What population group do you belong to?

Black African
Coloured
Indian/Asian
White
Prefer not to say
Other

A15. [SOUTH AFRICA ONLY] How do you describe your sexuality

Gay
Homosexual/
Bisexual
Heterosexual
Other (please say what _____________)
Don't know

A16 [SHOW IF KENYA]. How do you describe your sexuality in Swahili?

Basha
Hanithi
Kuchu
Kuruzi
Msago
Msenge
Shoga
Other (please say what ______________)

A16. What sex were you assigned at birth (e.g. on your original birth certificate)?

Male
Female
Prefer not to answer

A17. How do you currently describe your gender?

Male
Female
Transgender
I do not identify as male, female or transgender

A18. What is your current marital status?

Married / civil union / legal partnership

Single/divorced/widowed **[SKIP TO NEXT SECTION]**

A19. What is the gender of your spouse?

Male

Female

Transgender

Other – say what

B. SOCIAL: FRIENDSHIPS

We now want you to answer some questions about other men who have sex with men (MSM) who you know.

B1. How many other MSM do you know whom you have had a conversation with in the past month? By 'know', we mean someone who knows your name and you know theirs, and by 'had a conversation with', we mean either in person, on the phone, using SMS or online.

[enter number]

B2. How many of these [B5] men know each other?

All or almost all of them know each other

Most of them know each other [around **<ROUND[B5*.75]>** of them]

About half of them know each other [around **<ROUND[B5*.5]>** of them]

Some, but not the majority, know each other [around **<ROUND[B5*.25]**> of them]

Very few or none of them know each other

B3. How many of these [B1] men have you met in person?

[enter number]

B4. How many of these [B3] men are 18 years of age or older?

[enter number]

B5. How many of these [B4] men live in the same city as you?

[enter number]

B6. How many of these [B5] men have you seen in the past two weeks?

[enter number]

B7. How many of these [B6] men are over 30 years of age?

[enter number]

The next set of questions asks about how you meet and socialise with other men that have sex with men.

B8. When did you last visit any of these places to socialise with other MSM?

	Visited in the last month	Visited in the last year, but not in last month	Visited more than a year ago	Never visited for this reason
Outdoor meeting place e.g. the park, the street, the beach	◯	◯	◯	◯
Bar or club	◯	◯	◯	◯
Private house e.g. your house or a friends house	◯	◯	◯	◯
Sauna, bathhouse or sex party	◯	◯	◯	◯
Community space for MSM e.g. drop in centre, MSM organisation facilities	◯	◯	◯	◯

B9. When did you last use a website or mobile app to socialise with other MSM?

Never [**jump to B12**]
In the last month
In the last year
More than one year ago

B10. Which of the following internet services have you used to socialise with MSM in the last month [tick all that apply]?

2go ◯
Adam4Adam ◯
Badoo ◯
Dating Buzz ◯
Facebook ◯
Gay Radar ◯
Gay.com ◯
Gaydar ◯
Gayxchange ◯
Get Male ◯
Grindr ◯
Guy Spy ◯
Hornet ◯
Instagram ◯
iPlay ◯
Mambaonline ◯
Manhunt ◯
ManToManPlus ◯
Men2Men ◯
Radar ◯
Red Velvet ◯
Scruff ◯
Sex Trader South Africa ◯
Skype ◯
Snapchat ◯
Twitter ◯
Twoo ◯
WeChat ◯
Whatsapp ◯

MISTER ◯ Other ________

Hookups ◯ Planet Romeo ◯

B11. On the 1st January this year, were you registered as a user of the following services?

[SHOW IF COUNTRY=ALL]

B11.a. Grindr

Yes
No

B11.b. Planet Romeo

Yes
No

B11.c. Hornet

Yes
No

[SHOW IF COUNTRY=SOUTH AFRICA]

B11.d. Mamba Online

Yes
No

[If yes to B11a] B11.e. On January 1st this year, how many profiles do you have on Grindr?

[Enter number]

[If yes to B11b] B11.f. On January 1st this year, how many profiles do you have on Planet Romeo?

[Enter number]

[If yes to B11c] B11.f. On January 1st this year, how many profiles do you have on Hornet?

[Enter number]

[If yes to B11d] B11.g.On January 1st this year, how many profiles do you have on Mambo Online?

[Enter number]

B12. Have you ever visited or received information from a community organisation or support group for men who have sex with men?

No **[JUMP TO NEXT SECTION]**
Yes
Don’t know/not sure **[JUMP TO NEXT SECTION]**

B13. Which of the following gay/LGBT organisations or support groups have you VISITED in the past year?

[SHOW IF COUNTRY=SOUTH AFRICA]

SOHACA
(ANOVA)Health4Men
LGBTI
ACTIVATE
GALA
Other …

[SHOW IF COUNTRY=KENYA]

ISHTAR
GALCK
HOYMAS

C. SOCIAL SUPPORT

The next set of questions are about social support - or how much support you get from any partner/s, friends and family.

Please indicate how much you agree or disagree with the following statements

	Very strongly disagree	Strongly disagree	Mildly disagree	Neutral	Mildly agree	Strongly agree	Very strongly agree
**C1. There is a special person who is around when I am in need**							
	○	○	○	○	○	○	○
**C2. There is a special person with whom I can share joys and sorrows**							
	○	○	○	○	○	○	○
**C3. My family really tries to help me**							
	○	○	○	○	○	○	○
**C4. I get the emotional help & support I need from my family**							
	○	○	○	○	○	○	○
**C5. I have a special person who is a real source of comfort to me**							
	○	○	○	○	○	○	○
**C6. My friends really try to help me**							
	○	○	○	○	○	○	○
**C7. I can count on my friends when things go wrong**							
	○	○	○	○	○	○	○
**C8. I can talk about my problems with my family**							
	○	○	○	○	○	○	○
**C9. I have friends with whom I can share my joys and sorrows**							
	○	○	○	○	○	○	○
**C10. There is a special person in my life who cares about my feelings**							
	○	○	○	○	○	○	○
**C11. My family is willing to help me make decisions**							
	○	○	○	○	○	○	○
**C12. I can talk about my problems with my friends**							

	○	○	○	○	○	○	○

D. SOCIAL: DISCLOSURE & DISCRIMINATION DUE TO SEXUALITY

The next set of questions are about telling people about having sex with other men and any problems this might have caused you.

Please play close attention to the questions in this section. Some ask you to think about the last 12 months, while others ask you to think about your whole life (ever).

D1. Have you ever felt excluded from family activities because you have sex with men?

Yes
No

D2. Have you ever felt that family members have made discriminatory remarks or gossiped about you because you have sex with men?

Yes
No

D3. In general, do you try to keep it hidden from your FAMILY that you have sex with men?

I try very hard to hide it
Try somewhat to hide it
I don't try to hide it, but I don't talk about it
I openly talk about it
Not applicable

D4. In general, do you try to keep it hidden from your FRIENDS that you have sex with men?

I try very hard to hide it
Try somewhat to hide it
I don't try to hide it, but I don't talk about it
I openly talk about it
Not applicable

D5. Have you ever felt rejected by your friends because you have sex with men?

Yes
No

D6. In general, do you try to keep it hidden from HEALTH CARE WORKERS that you have sex with men?

I try very hard to hide it
Try somewhat to hide it
I don't try to hide it, but I don't talk about it
I openly talk about it
Not applicable

D7. In the last 12 months, have you felt afraid to go to health care services because you worry someone may learn you have sex with men?

Yes
No

D8. In the last 12 months, have you avoided going to health care services because you worry someone may learn you have sex with men?

Yes
No

D9. In the last 12 months, have you felt that you were not treated well in a health centre because someone knew that you have sex with men?

Yes
No

D10. In the last 12 months, have you heard health care providers gossiping or laughing about you because you have sex with men?

Yes
No

D11. In the last 12 months, have you felt that the police refused to protect you because you have sex with men?

Yes
No

D12. In the last 12 months, have you felt scared to be in public places because you have sex with men?

Yes
No

D15. In the last 12 months, have you been blackmailed by someone because you have sex with men?

Yes
No

D17. In the last 12 months, has someone ever physically hurt you (pushed, shoved, slapped, hit, kicked, choked or otherwise physically hurt you) due to the fact that you have sex with men?

Yes
No **[JUMP TO D20]**

D18. Who did this? [Tick all that apply]

A stranger
A family member
A member of the police or a public official
A health provider
A partner

A sex client
Other

D20. In the last 12 months, have you been forced to have sex when you did not want to? (By forced, we mean physically forced, coerced to have sex, or penetrated with an object, when you did not want to) due to the fact that you have sex with men?

Yes
No **[JUMP TO D24]**

D21. Who did this? [Tick all that apply]

A stranger
A family member
A member of the police or a public official
A health provider
A partner
A sex client
Other

D24. In the last 12 months, have you been threatened with detention or arrest by a member of the police or public official due to the fact you have sex with men?

Yes
No

D26. Have you ever been arrested or imprisoned because you have sex with men?

Yes
No

E. SEXUAL BEHAVIOUR

We would now like to ask you about your recent sexual behaviour. By sex we mean any genital contact - including masturbation (wanking, fingering); oral sex (sucking/blowjobs, licking, rimming); vaginal or anal intercourse (fucking).

E1. In the last 3 months, have you had sex with a man?

Yes

No

E1a. [If E1 = yes] How many different men have you had sex with in the last 3 months?

[Enter number]

E2. In the last 3 months, what type of anal sex have you engaged in with men?

Receptive anal sex only (‘bottom’)

Insertive anal sex only (‘top’)

Both receptive and insertive anal sex (versatile)

None of the above

E3 [if E2= receptive or both] In the last 3 months, how often did you use condoms when you were the receptive partner / were bottom for anal sex?

Always

Most of the time

Some of the time

Rarely

Never

E4. [if E2= Insertive or both] In the last 3 months, how often did you use condoms when you were the insertive partner / were top for anal sex?

Always

Most of the time

Some of the time

Rarely

Never

E5. In the last 3 months, what type of oral sex have you engaged in with men?

Receptive oral sex only (I gave a blow job)

Insertive oral sex only (I was sucked off)

Both ways

None of the above

E6. In the last 12 months, have any men paid you in return for sex? By payment we mean he gave you money, gifts or favours in return for sex.

Yes
No

E7. In the last 12 months, have you paid a man in return for sex with him? By payment we mean you gave him money, gifts or favours in return for sex.

Yes
No

E8. In the last 12 months, have any of your male partners every tried to hurt you? By this we mean pushing, holding you down, hitting you with his fist, kicking, attempting to strangle, attacking with a knife, gun or other weapon

Yes
No

E9. In the last 12 months, have any of your male partners used physical force or verbal threats to force you to have sex when you did not want to?

Yes
No

E10. In the last 3 months have you had sex with a woman?

Yes

No [JUMP TO NEXT SECTION]

E10a. How many women have you had sex with in the 3 months?

[enter number]

E11. In the last 3 months, what type of sex have you engaged in with women?

Vaginal sex

Anal sex

Both

Neither

E12. [if E11= vaginal or both] In the last 3 months, how often did you use condoms when you had vaginal sex with a female partner?

Always

Most of the time

Some of the time

Rarely

Never

E13 [if E11=anal or both] In the last 3 months, how often did you use condoms when you had anal sex with a female partner?

Always

Most of the time

Some of the time

Rarely

Never

E14. In the last 12 months, have any women paid you in return for sex? By payment we mean she gave you money, gifts or favours in return for sex.

Yes

No

E15. In the last 12 months, have you paid a woman in return for sex with her? By payment we mean you gave her money, gifts or favours in return for sex.

Yes

No

EX. SEXUAL BEHAVIOUR: PARTNERSHIP DYADS

We now want you to think about the last FOUR people you had sex with. We want to ask you a set of questions about each of these FOUR people. Choose a nickname or set of initials to help identify each one. These do not need to be real nicknames or initials, just something that will help keep track of who we are asking about.
Remember that by sex we mean any genital contact - including masturbation (wanking, fingering); oral sex (sucking/blowjobs, licking, rimming); vaginal or anal intercourse (fucking).

Partner #1:
Who was the last person you had sex with? (Write in a nickname, first name or initials)
[Enter initials]

Partner # 2:
Before (Partner #1) who was the last person you had sex with? (Write in a nickname, first name or initials)
[Enter initials]

Partner #3:
Before (Partner #2) who was the last person you had sex with? (Write in a nickname, first name or initials)
[Enter initials]

Partner #4:
Before (Partner #3) who was the last person you had sex with? (Write in a nickname, first name or initials)
[Enter initials]

Partner #1

The following set of questions are about the first partner you mentioned.

There are three main routes through this section of the survey, depending on whether they are (1) a casual, one-off partner, (2) a regular sexual partner who they expect to have sex with again in the future, and (3) a partner who was regular but who they do not expect to have sex with again in the future. The online survey hosting system will automatically route people through the survey based on prior responses. For this Word version, all three routes are presented in turn. Revisions were necessary to this section following piloting. The questions remain largely the same as our previous submission, but have been re-ordered for clarity of completion and in anticipation of multiple partner types.

ROUTE 1: A CASUAL, ONE-OFF SEXUAL PARTNER

What is the most recent date [closest to today] you had sex with [partner #1]? Please make your best guess if you cannot remember exactly.

[enter date]

What is the gender of [partner #1]

Male
Female
Transgender
Don't know

How old was [partner #1] when you last had sex with them? Please make your best guess if you are unsure.

[enter age]

How did you first meet [partner #1]?

Through friends
Through family
At school
At work
At a bar or club
At a private party
On a social networking site - say which one
Other - say where you met
I don't remember

How many times have you ever had sex with [partner #1]

Only once
More than once

Do you expect to have sex with [partner #1] again in the future?

Yes

No

Did you give [partner #1] money, gifts or favours in exchange for sex with you?

No
Yes

Did [partner #1] give you money, gifts or favours in exchange for sex with you?

No
Yes

EX28[i] [if EX3=male]
What type of sex have you ever had with [Partner #1]? [tick all that apply]

Receptive anal sex (I was bottom)
Insertive anal sex (I was top)
Receptive oral sex (I gave a blow job)
Insertive oral sex (I was sucked off)

EX29A[i] [if EX3=male & EX28 = receptive anal sex]
When you had receptive anal sex / was bottom with [Partner #1], was this with or without a condom?

With a condom
Without a condom

EX29B[i] [if EX3=male & EX28 = insertive anal sex]
When you had insertive anal sex / was top with [Partner #1], was this with or without a condom?

With a condom
Without a condom

EX30[i] [if EX3=female]
What type of sex have you ever had with [Partner #1]? [tick all that apply]

Vaginal sex
Anal sex
Oral sex

EX31A[i] [if EX3= female & EX28 = vaginal sex]
When you had vaginal sex with [Partner #1], was this with or without a condom?

With a condom
Without a condom

EX31B[i] [if EX3= female & EX28 = anal sex]
Last time you had anal sex with [Partner #1], was this with or without a condom?

With a condom
Without a condom

EX33[i] Has [Partner #1] shared his/her HIV status with you?

No **[JUMP TO E35[i]]**

Yes

I do not know

EX34[i] What is [Partner #1]'s HIV status?

HIV-positive [they had HIV infection]

HIV-negative [they did not have HIV infection]

I do not know

SKIP TO E36[i]

EX35[i] What did you believe [Partner #1]'s HIV status to be at the time you had sex with them?

I think HIV-positive

I think HIV-negative

I do not know

ROUTE 2: A REGULAR SEXUAL PARTNER WHO THEY EXPECT TO HAVE SEX WITH AGAIN IN THE FUTURE

What is the most recent date [closest to today] you had sex with [partner #1]? Please make your best guess if you cannot remember exactly.

[enter date]

What is the gender of [partner #1]

Male

Female

Transgender

Don't know

How old was [partner #1] when you last had sex with them? Please make your best guess if you are unsure.

[enter age]

How did you first meet [partner #1]?

Through friends

Through family

At school

At work

At a bar or club

At a private party

On a social networking site - say which one

Other - say where you met
I don’t remember

How many times have you ever had sex with [partner #1]

Only once
More than once

Do you expect to have sex with [partner #1] again in the future?

Yes
No

EX20A[i] [if EX19i=YES] Which of the following best describes your current relationship with <EX1[i]>?

We are married / in a civil partnership with each other
We are going steady / regular partners (i.e. long term boyfriend or girlfriend)
We are friends who occasionally have sex
We just meet for sex

EX21A[i] [if EX19i=YES] Do you live with them?

Yes
No

EX22A[i] [if EX19i=YES] Are you in love with <EX1[i]>?

Yes, very much
Yes, a little
No

EX24A[i] [if EX19i=YES] Are you dependent on <EX1[i]> for income, money or somewhere to stay?

Yes, very much
Yes, a little
No

EX26[i]. Do you give them money, gifts or favours in exchange for sex with you?

No
Yes

EX27[i]. Do they give you money, gifts or favours in exchange for sex with you?

No
Yes

EX28[i] [if EX3=male]
What type of sex have you ever had with [Partner #1]? [tick all that apply]

Receptive anal sex (I was bottom)
Insertive anal sex (I was top)
Receptive oral sex (I gave a blow job)
Insertive oral sex (I was sucked off)

EX29A[i] [if EX3=male & EX28 = receptive anal sex]

Last time you had receptive anal sex / was bottom with [Partner #1], was this with or without a condom?

With a condom

Without a condom

EX29B[i] [if EX3=male & EX28 = insertive anal sex]

Last time you had insertive anal sex / was top with [Partner #1], was this with or without a condom?

With a condom

Without a condom

EX30[i] [if EX3=female]

What type of sex have you ever had with [Partner #1]? [tick all that apply]

Vaginal sex

Anal sex

Oral sex

EX31A[i] [if EX3= female & EX28 = vaginal sex]

Last time you had vaginal sex with [Partner #1], was this with or without a condom?

With a condom

Without a condom

EX31B[i] [if EX3= female & EX28 = anal sex]

Last time you had anal sex with [Partner #1], was this with or without a condom?

With a condom

Without a condom

EX33[i] Has [Partner #1] shared his/her HIV status with you?

No **[JUMP TO E35[i]]**

Yes

I do not know

EX34[i] What is [Partner #1]'s HIV status?

HIV-positive [they had HIV infection]

HIV-negative [they did not have HIV infection]

I do not know

SKIP TO E36[i]

EX35[i] What do you believe [Partner #1]'s HIV status to be?

I think HIV-positive

I think HIV-negative

I do not know

EX36[i] Have you and [Partner #1] ever accompanied each other to any of the following [check any that apply]

Counselling and testing for HIV
Appointment at a sexual health clinic
Appointment at an HIV treatment clinic
A health information session or event for MSM
Attended a social event arranged by an MSM organisation
NO, none of the above

ROUTE 3: A FORMERLY REGULAR SEXUAL PARTNER WHO THEY DO NOT EXPECT TO HAVE SEX WITH AGAIN IN THE FUTURE

What is the most recent date [closest to today] you had sex with [partner #1]? Please make your best guess if you cannot remember exactly.

[enter date]

What is the gender of [partner #1]

Male
Female
Transgender
Don't know

How old was [partner #1] when you last had sex with them? Please make your best guess if you are unsure.

[enter age]

How did you first meet [partner #1]?

Through friends
Through family
At school
At work
At a bar or club
At a private party
On a social networking site - say which one
Other - say where you met
I don't remember

How many times have you ever had sex with [partner #1]

Only once
More than once

Do you expect to have sex with [partner #1] again in the future?

Yes
No

EX20B[i] [if EX19i=NO] Which of the following best describes the relationship you had with <EX1[i]>?

We were married / in a civil partnership with each other
We were going steady / regular partners (i.e. long term boyfriend or girlfriend)
We were friends who occasionally had sex
We just met for sex

EX21B[i] [if EX19i=NO & EX18i=YES] When you were in a relationship, did you live with <EX1[i]>?

Yes
No

EX22B[i] [if EX19i=NO & EX18i=YES] When you were in a relationship, were you in love with <EX1[i]>?

Yes, very much
Yes, a little
No

EX24B[i] [if EX19i=NO] Were you dependent on <EX1[i]> for income, money or somewhere to stay?

Yes, very much
Yes, a little
No

EX26[i]. Did you give them money, gifts or favours in exchange for sex with you?

No
Yes

EX27[i]. Did they give you money, gifts or favours in exchange for sex with you?

No
Yes

EX28[i] [if EX3=male]
What type of sex have you ever had with [Partner #1]? [tick all that apply]

Receptive anal sex (I was bottom)
Insertive anal sex (I was top)
Receptive oral sex (I gave a blow job)
Insertive oral sex (I was sucked off)

EX29A[i] [if EX3=male & EX28 = receptive anal sex]
Last time you had receptive anal sex / was bottom with [Partner #1], was this with or without a condom?

With a condom
Without a condom

EX29B[i] [if EX3=male & EX28 = insertive anal sex]
Last time you had insertive anal sex / was top with [Partner #1], was this with or without a condom?

With a condom
Without a condom

EX30[i] [if EX3=female]

What type of sex have you ever had with [Partner #1]? [tick all that apply]

Vaginal sex

Anal sex

Oral sex

EX31A[i] [if EX3= female & EX28 = vaginal sex]

Last time you had vaginal sex with [Partner #1], was this with or without a condom?

With a condom

Without a condom

EX31B[i] [if EX3= female & EX28 = anal sex]

Last time you had anal sex with [Partner #1], was this with or without a condom?

With a condom

Without a condom

EX33[i] Has [Partner #1] shared his/her HIV status with you?

No **[JUMP TO E35[i]]**

Yes

I do not know

EX34[i] What is [Partner #1]'s HIV status?

HIV-positive [they had HIV infection]

HIV-negative [they did not have HIV infection]

I do not know

SKIP TO E36[i]

EX35[i] What did you believe [Partner #1]'s HIV status to be when you last had sex with them?

I think HIV-positive

I think HIV-negative

I do not know

EX36[i] Have you and [Partner #1] ever accompanied each other to any of the following [check any that apply]

Counselling and testing for HIV

Appointment at a sexual health clinic

Appointment at an HIV treatment clinic

A health information session or event for MSM

Attended a social event arranged by an MSM organisation

NO, none of the above

To finish off this set we are going to ask you a few questions about whether any of the partners you just described know each other.

EX37 [IF >1 MALE] As far as you know, are any of these men sexually active with each other, as well as with you?

Yes
No **[SKIP TO NEXT SECTION]**
I don't know **[SKIP TO NEXT SECTION]**

EX38 [IF >1 MALE REPORTED] Please indicate which men are sexually active with each other

<EX1> & <EX2> [IF BOTH MALE]
<EX1> & <EX3> [IF BOTH MALE]
<EX1> & <EX4> [IF BOTH MALE]
<EX2> & <EX3> [IF BOTH MALE]
<EX2> & <EX4> [IF BOTH MALE]
<EX3> & <EX4> [IF BOTH MALE]

F. KNOWLEDGE & SELF EFFICACY REGARDING HIV & SEXUAL HEALTH

The next set of questions are about what you know about HIV and how easy you find it to manage your sexual health.

F1. The following statements are all TRUE. Did you know this already?

F1a. 'Effective treatment of HIV infection reduces the risk of HIV being transmitted'

- I knew this already
- I wasn't sure about this
- I didn't know this already
- I don't understand this

F1b. 'It is possible to catch HIV during anal sex with a man'

- I knew this already
- I wasn't sure about this
- I didn't know this already
- I don't understand this

F1c. 'It is possible to catch HIV even when you are the active partner (the 'top') during anal sex'

- I knew this already
- I wasn't sure about this
- I didn't know this already
- I don't understand this

F2. To what extent do you agree with the following statements?

	Strongly disagree	Mildly disagree	Neutral	Mildly agree	Strongly agree
**F2a. 'The sex I have is always as safe as I want it to be'**					
	○	○	○	○	○
**F2b. 'I can ensure condoms are used with a sexual partner if I want them to be used'**					
	○	○	○	○	○
**F2c. 'I sometimes have a problem getting condoms when I need them'**					
	○	○	○	○	○
**F2d. 'I sometimes have problems with condoms that do not fit properly'**					
	○	○	○	○	○
**F2e. 'I sometimes have a problem getting water-based lubricant when I need it'**					
	○	○	○	○	○

G. SEXUAL HEALTH & HIV

The next set of questions are about testing for HIV.

G1. What do you believe your current HIV status is today?

Negative (I do not think I have HIV)
Positive (I think I have HIV)
Not sure

G2. Have you ever taken an HIV test?

Yes
No **[jump to section G PART III]**

G3: In what month and year was your most recent HIV test?

[enter date MM/YYYY]

G4: Where did you take your most recent HIV test?

Public hospital or clinic
Private hospital or clinic
Community HIV testing service for the public
Community HIV testing service for MSM only
A place where I meet my friends (bar or club)
At home
Other [please specify]

G5. When you took your last HIV test, were you satisfied with the privacy of the service?

Very satisfied
Satisfied
Dissatisfied
Very dissatisfied
I don't remember / I did not think about it

G7. When you took your last HIV test, were you satisfied with the respect staff showed you?

Very satisfied
Satisfied
Dissatisfied
Very dissatisfied
I don't remember / I did not think about it

G8. What was the result of your most recent HIV test?

Negative (I did not have HIV at my last test)
Positive (I have HIV)
I don't know

SECTION G PART I: ABOUT BEING HIV POSITIVE

The next set of questions are about having diagnosed HIV.

G10. In what month and year were you first diagnosed HIV positive?

[enter date MMYY]

G11. When you were first diagnosed HIV positive, were you referred to a health care provider about your HIV infection or HIV related health? By 'health care provider' we mean a clinic at which you might see a doctor or clinical officer about your medical care.

Yes

No

G12. Since you were first diagnosed HIV positive, have you ever visited a health care provider about your HIV infection or HIV related health?

Yes **[JUMP TO G14]**

No

G13 Why did you not visit the health care provider after having been referred to them? (tick any that apply)

Negative attitude of clinic staff toward MSM

Negative attitude of clinic staff toward people living with HIV

Fear of being noticed going to the clinic

Long distance to get to the clinic

High cost of the clinic/tests

High cost of travel to the clinic

High cost of medication

Fear of medication side effects

Fear that my medication will be noticed

Did not believe the medication would work

Did not believe I needed the medication

Other reason [please specify________________]

G14. From the date that you learnt you were HIV positive, how soon did you first see a health care provider for HIV care?

On the same day as I learned my status

Within 2 weeks of learning my status

2 to 4 weeks after learning my status

1 to 3 months after learning my status

3 to 12 months after learning my status

Over one year after learning my status

ADDED Never seen health care provider about my HIV

G15. Where did you go the first time you visited a health care provider for this purpose?

A public hospital or clinic

A private hospital or clinic
A clinic just for men who have sex with men

ROUTINE HIV CARE

The next set of questions is about routine HIV care and support.

G16. When did you last see a health professional about your HIV care?

Within the last 6 months
Between 6 to 12 months ago
Between 1 to 2 years ago
More than 2 years ago

G17. Where did you go the last time you visited a health care provider for this purpose?

A public hospital or clinic
A private hospital or clinic
A clinic just for men who have sex with men

G18. The last time you visited a health care provider for HIV care, were you satisfied with the privacy of the service?

Very satisfied
Satisfied
Dissatisfied
Very dissatisfied
I don't remember / I did not think about it

G20. The last time you visited a health care provider for HIV care, were you satisfied with the respect staff showed you?

Very satisfied
Satisfied
Dissatisfied
Very dissatisfied
I don't remember / I did not think about it

G22. Have you ever received a test to see how well your immune system is functioning. This is called a CD4 test?

Yes
No
Not sure

G22a. [If G22 = yes] When did you last receive a CD4 count test result?

Within the last 6 months
Between 6 to 12 months ago
Between 1 to 2 years ago
More than 2 years ago

G23. [If G22 = yes] What was your CD4 count the last time this was tested?

More than 500

Between 350-500

Under 350

I was told the result, but I do not remember

I was told the result, but I did not understand it

I was not told the result

G24. Have you ever received a test to measure the amount of HIV infection in your blood? This is called a viral load test.

Yes

No

Not sure

G24a. [If G24 = yes] When did you last receive a viral load test result?

Within the last 6 months

Between 6 to 12 months ago

Between 1 to 2 years ago

More than 2 years ago

G25. [If G24 = yes] What was the result of your viral load the last time it was tested?

Undetectable

Detectable

I was told the result, but I do not remember

I was told the result, but I did not understand it

I was not told the viral load test result

HIV TREATMENT (ART)

The next set of questions is about taking anti-HIV treatments (ART, HAART).

G26. Have you ever started taking antiretroviral therapy (sometimes called ART or HAART) for your HIV infection?

Yes

No **[JUMP TO G28]**

G27. You have said you first learned of your HIV infection in <G8 MM YY>. How soon after this did you start ART?

On the same day as I learned my status

Within 2 weeks after learning my status

2 to 4 weeks after learning my status

1 to 3 months after learning my status

3 to 12 months after learning my status

Over one year after learning my status

[JUMP TO SECTION G PART IV]

G28. Has your healthcare provider suggested you start antiretroviral treatment (ART)?

Yes

No **[JUMP TO SECTION G PART IV]**

G29. What factors made you decide not to start antiretroviral treatment (ART)? [Tick all that apply]

Negative attitude of clinic staff toward MSM

Negative attitude of clinic staff toward people living with HIV

Fear of being noticed going to the clinic

Long distance to get to the clinic

High cost of the clinic/tests

High cost of travel to the clinic

High cost of medication

Fear of medication side effects

Fear that my medication will be noticed

Did not believe the medication would work

Did not believe I needed the medication

Other reason [please specify________________]

[JUMP TO SECTION G PART IV]

G30. Are you currently taking antiretroviral treatment (ART)?

Yes

No

[If G30 = no] When did you stop taking antiretroviral treatment (ART)?

Within the last 6 months

Between 6 to 12 months ago

Between 1 to 2 years ago

More than 2 years ago

[If G30 = no] G32. What factors made you decide to stop taking antiretroviral treatment? [Tick all that apply]

Negative attitude of clinic staff toward MSM
Negative attitude of clinic staff toward people living with HIV
Fear of being noticed going to the clinic
Long distance to get to the clinic
High cost of the clinic/tests
High cost of travel to the clinic
High cost of medication
Fear of medication side effects
Fear that my medication will be noticed
Did not believe the medication was working
Did not believe I needed the medication any more
Other reason [please specify_______________]

[If G30 = yes] G31. Many patients find it difficult to take all of their HIV medication exactly as prescribed. How many doses of your HIV medication did you miss in the last 7 days?

[Enter number of doses]

SECTION G PART II: ABOUT BEING HIV NEGATIVE

The next set of questions is about testing negative for HIV.

G33. In the last 12 months, how many times have you taken an HIV test?

[enter a number]

G34. If you decided to take an HIV test again, where would you prefer to do the test?

Public hospital or clinic
Private hospital or clinic
Community HIV testing service for the public
Community HIV testing service for MSM only
A place where I meet my friends (e.g. bars or clubs)
At home

G35. If you decided to take an HIV test again, who would you prefer to perform the test?

Doctor or clinical officer
Nurse
Counsellor
MSM community worker
Me [i.e. self-test]

[JUMP TO SECTION G PART IV]

SECTION G PART III: ABOUT NEVER TESTING FOR HIV

The next set of questions are about never having tested for HIV.

G36. Please give any reasons why you never tested for HIV before?

[enter text]
It is not important to me to know
I expect I have the same status as my partner
I do not know where to get tested
I may not be treated with respect at the clinic
I am afraid that I might have HIV
I am afraid of being treated differently if I take a test
I am afraid of being treated differently if I have HIV
It would cause problems in my relationship
I have no reason to think I have HIV
Other reason (say what_______________)

G37. How confident are you that you could get a test for HIV if you wanted one in the future?

Very confident
Quite confident
A little confident
Not at all confident
I don't know

G38. If you decided to take an HIV test in the future, where would you prefer to do the test?

Public hospital or clinic
Private hospital or clinic
Community HIV testing service for the public
Community HIV testing service for men who have sex with men only
A place where I meet my friends (e.g. bars or clubs)
At home

G39. If you decided to take an HIV test in the future, who would you prefer to perform the test?

Doctor or clinical officer
Nurse
Counsellor
MSM community worker
Me [i.e. self-test]

SECTION G PART IV: OTHER SEXUALLY TRANSMITTED INFECTIONS

The next set of questions are about the symptoms of certain sexually transmitted infections.

G40. In the last 12 months, have you had a discharge from the penis or pain when you pass urine?

Yes

No **[JUMP to G42]**

G41. Do you have these symptoms today?

Yes

No

G42. In the last 12 months, have you had a discharge from the anus or severe pain during anal sex?

Yes

No **[JUMP to G44]**

G43. Do you have these symptoms today?

Yes

No

G44. In the last 12 months, have you noticed any sores on the penis or around the anus?

Yes

No

[IF A16 = A17 PAST TO NEXT SECTION]

SECTION G PART V: TRANSGENDER SEXUAL HEALTH ACCESS

IF DISCREPENCY BETWEEN ANSWER TO A16 AND A17, OR IF TRANS IDENTIFYING IN A17

The next of questions is about access to - and use of - transgender services.

G45. How confident are you that you could access counselling concerning your gender?

Very confident
Quite confident
A little confident
Not at all confident
I don't know

G46. Do you currently use hormone or hormone-blocking therapies?

Yes
No **[SKIP TO G48]**

G47. From where do you access these therapies?

Public hospital or clinic
Private hospital or clinic
Purchased directly from a pharmacy
Purchased over the internet
Obtained from friends

[SKIP TO G49]

G48. How confident are you that you could access hormone or hormone-blocking therapies in <COUNTRY> if you wanted them?

Very confident
Quite confident
A little confident
Not at all confident
I don't know

G49. Have you had any surgery related to your gender [PILOT: REQUIRES ACCEPTABLE & UNDERSTANDABLE LOCAL TERM]?

Yes
No **[SKIP TO G51]**

G50. Where were you able to access these services?

Public hospital in this country
Private hospital in this country
Hospital in another country

[JUMP TO NEXT SECTION]

G51. How confident are you that you could access surgery related to your gender

Very confident
Quite confident
A little confident
Not at all confident
I don't know

H. Post Exposure Prophylaxis (PEP)

The next set of questions is about Post Exposure Prophylaxis - which is also known as PEP.

H1. The following statement is true. Did you know this already?
Post exposure prophylaxis (PEP) is a one-month course of pills that may stop someone from becoming infected with HIV if they are exposed to the virus (such as by having sex without condoms). PEP needs to be started as soon as possible AFTER an HIV risk.

I knew this already
I wasn't sure about this
I didn't know this already
I don't understand this

H2. Have you ever tried to get PEP?

Yes
No
Don't know

H3. [If yes to H2] Have you ever taken PEP?

Yes
No
Don't know

H4. [If yes to H3] For how many days did you take PEP? (If you've taken it more than once, think about the last time you took it)

[enter number]

H5. [IF G1 = NEGATIVE or NOT SURE] If you thought you had been exposed to HIV would you know where to get PEP?

Yes
No
Don't know

H. Pre Exposure Prophylaxis (PrEP)

The next set of questions is about Pre Exposure Prophylaxis - which is also known as PrEP.

H6. The following statement is true. Did you know this already?

Pre exposure prophylaxis (PrEP) involves someone who does not have HIV taking a pill on an on-going basis to prevent them getting HIV. Most people who use PrEP take a pill everyday. PrEP needs to be taken BEFORE sex for it to be effective.

I knew this already
I wasn't sure about this
I didn't know this already
I don't understand this

PrEP is different from PEP. PEP is taken AFTER a risk and PrEP is taken BEFORE.

H7. Have you ever tried to get PrEP?

Yes
No **[JUMP TO H10**]
Don't know **[JUMP TO H10]**

H8. Have you ever been offered PrEP?

Yes
No
Not sure/don't know

H8. Have you ever taken PrEP?

Yes, and I am still using it
Yes, but I stopped taking it
No **[JUMP TO H10]**
Don't know **[JUMP TO H10]**

H9. Where did you access your PrEP?

A doctor at a public hospital or clinic
A doctor at a private hospital or clinic
At a community based organization
From a dedicated website (say which one)
Somewhere else (say where)

[JUMP TO NEXT SECTION]

H10. [IF G1 = NEGATIVE or NOT SURE] If PrEP was made available to you how likely do you think that you would use it?

Very likely

Quite likely
Not sure
Not very likely
Very unlikely

H11. If PrEP was made available to you, where would you prefer to access it? ~~[Tick one]~~

At a public hospital or clinic
At a private hospital or clinic
At a community based organization
From a pharmacy
From a dedicated website
Somewhere else (say where)

SECTION I. ALCOHOL USE

The next set of questions are about drinking alcohol.

I1. How often do you have a drink containing alcohol?

Never

Monthly

2-4 times a month

2-3 times a week

4 or more times a week

I2. How many drinks containing alcohol do you have on a typical day when you are drinking?

1 or 2

3 or 4

5 or 6

7 to 9

10 or more

I3. How often do you have six or more drinks on one occasion?

Never

Less than monthly

Monthly

Weekly

Daily or almost daily

I4. How often during the last year have you found that you were not able to stop drinking once you had started?

Never

Less than monthly

Monthly

Weekly

Daily or almost daily

I5. How often during the last year have you failed to do what was normally expected of you because of drinking?

Never

Less than monthly

Monthly

Weekly

Daily or almost daily

I6. How often during the last year have you needed a first drink in the morning to get yourself going after a heavy drinking session?

Never

Less than monthly

Monthly

Weekly

Daily or almost daily

I7. How often during the last year have you had a feeling of guilt or remorse after drinking?

Never

Less than monthly

Monthly

Weekly

Daily or almost daily

I8. How often during the last year have you been unable to remember what happened that night before because of your drinking?

Never

Less than monthly

Monthly

Weekly

Daily or almost daily

I9. Have you or someone else been injured because of your drinking?

No

Yes, but not in the last year

Yes, during the last year

I10. Has a relative, friend, doctor or other health care worker been concerned about your drinking or suggested you cut down?

No

Yes, but not in the last year

Yes, during the last year

SECTION J. SUBSTANCE USE

The next set of questions are about your use of tobacco and other drugs ...

J1. Please indicate when you have used any of the following substances:

**Substance**	**Never**	**Within the last month**	**Within the last year but not the last month**	**More than one year ago**
Tobacco	◯	◯	◯	◯
Cannabis (grass, weed, herb, ndom, bhang, ganja, dagga, zol)	◯	◯	◯	◯
Khat (miraa, veve, mogoka)	◯	◯	◯	◯
Ecstasy (E, umgwinyo, happy pill, disco biscuit, Adam)	◯	◯	◯	◯
Amphetamine (speed, gavana)	◯	◯	◯	◯
Crystal methamphetamine (crystal, ice, tina, meth, taptap, crank)	◯	◯	◯	◯
Heroin (smack, mud, brown sugar)	◯	◯	◯	◯
Mephedrone (meow meow, plant food, bubbles, kitty cat)	◯	◯	◯	◯
GHB/GBL (G, liquid ecstasy, soap)	◯	◯	◯	◯
Cocaine or Crack cocaine (rock)	◯	◯	◯	◯
Rohypnol (mchele, roofies, forget pill)	◯	◯	◯	◯
Poppers (liquid gold)	◯	◯	◯	◯
Benzene	◯	◯	◯	◯

K. MENTAL HEALTH

The next set of questions are about how you have been feeling in the last two weeks.

In the last two weeks how often have you been bothered by any of the following problems:

	Not at all	Several days	More than half the days	Nearly every day
**K1. Little interest or pleasure in doing things**				
	○	○	○	○
**K2. Feeling down, depressed or hopeless**				
	○	○	○	○
**K3. Trouble falling or staying asleep, or sleeping too much**				
	○	○	○	○
**K4. Feeling tired or having little energy**				
	○	○	○	○
**K5. Poor appetite or overeating**				
	○	○	○	○
**K6. Feeling bad about yourself, or that you are a failure, or have let yourself or your family down**				
	○	○	○	○
**K7. Trouble concentrating on things, such as reading the newspaper or watching television**				
	○	○	○	○
**K8. Moving or speaking so slowly that other people could have noticed? Or the opposite – being so fidgety or restless that you have been moving around a lot more than usual**				
	○	○	○	○
**K9. Thoughts that you would be better off dead or of hurting yourself in some way**				
	○	○	○	○

K10. If you checked off any of the problems above, how difficult have these problems made it for you to do your work, take care of things at home, or get along with other people?

Not difficult at all
Somewhat difficult
Very difficult
Extremely difficult

L. POPULATION ESTIMATORS

We now wish to ask whether or not you have used particular services recently. The answers to these questions will help us to estimate the number of MSM in the local area. No personal information will be passed on to or requested from any of these services to make this estimate.

[SHOW IF SITE = NAIROBI]

L1. At [reference date], were you friends with the Facebook group 'ISHTAR-MSM'?

Yes
No
Do not know

L2. Did you have an appointment at [Liverpool VCT or ISHTAR clinic] between [enter reference period]

Yes
No
Do not know

[SHOW IF SITE = SOUTH AFRICA]

L1. At [reference date], were you registered as a member of the Facebook group 'Black Men Bold and the Beautiful'?

Yes
No
Do not know

L2. At [reference date], were you registered as a member of the Facebook group 'Johannesburg Gays'?

Yes
No
Do not know

L3. At [reference date], were you registered as a member of the Facebook group 'Soweto Gays'?

Yes
No
Do not know

L4. At [reference date] were you following 'We the Brave' on Facebook?

Yes
No
Don't know

L5. At [reference date] were you following 'Health 4 Men' on Facebook?

Yes
No

Don’t know

L6. Did you visit an ANOVA Health 4 Men clinic between [enter reference period]

ANOVA
HEALTH INSTITUTE

Yes
No
Do not know

[If L6 = yes] L7. Which ANOVA Health 4Men clinic did you visit?

[List of available clinics]
Zola
Chiawelo
Yeoville
Other__________

M. HAPPINESS WITH SEX

The last set of questions ask how happy you are with your sex life, and what might make it better.

M1. How happy are you with your sex life right now?

Very happy
Quite happy
Not sure/don't know
Quite unhappy
Very unhappy

M2. What one thing would help to make your sex life better?

[enter text]

M3. Who is the sexiest man on the planet?

[enter text]

Thank you very much for taking this survey. Your response is very important to us.

If you feel you may have made any mistakes which you want to go back and correct, please mention this to the researcher who will help you.

If the survey has made you think of questions about your health or about this research study, please ask the researcher..

TRANSFORM Survey Instrument: Coupon Issue Module

Date today: [year/months/day]

Site name:

Study Staff name:

Participant ID number:

Coupon Number:

Return visit

B1. How many other MSM do you know whom you have had a conversation with in the past month? By 'know', we mean someone who knows your name and you know theirs, and by 'had a conversation with', we mean either in person, on the phone, using SMS or online.

[enter number]

B2. How many of these [B1] men have you met in person?

[enter number]

B3. How many of these [B2] men are 18 years of age or older?

[enter number]

Q9. How many of these [B3] men live in [Johannesburg'/Nairobi]?

[enter number]

Q10. How many of these [B4] men have you seen in the past two weeks?

[enter number]

YOUR COUPON DISTRIBUTION (REFUSALS)

We want to ask you about the people who did NOT accept a coupon from you.

Q11 **How many of the men you offered a coupon to, did not take one from you?**
[enter number]

Q12 **[For each of [Q11]**
Thinking about the first man you offered a coupon too, that refused to take it,
Why did that man not take a coupon from you? [tick all that apply]
He said that he had already participated in the study
He said that he had already received a coupon from someone else
He said that he was not an MSM
He said he was younger than 18 years old
He said he was not from the same city as me
He did not wish to participate in the study
He said that there was not enough money for participating
Other reason – say what
None of the above

YOUR COUPON DISTRIBUTION (ACCEPTED)

We now want to ask you about the people who did accept a coupon from you.

Q13 **How many men did you offer a coupon to who accepted it?**

[enter number 0-3]

IF Q13>0 Concerning the first person you gave a coupon to:

Q14i: Thinking about the first man you offered a coupon too, that took it:

Do you think this person would have given you a coupon if they had participated in the study before you?

Yes
No

Q14i: Still thinking about the first man you offered a coupon too, that took it:
Is this person over 30 years of age?
Yes
No
Don't know

Q14i: Still thinking about the first man you offered a coupon too, that took it:
What made you decide to give a coupon to this person? [Choose the most important reason]
He was the first eligible man I met
He is a close friend
He is my partner
He needed the money for participation
I thought he would benefit from the study
I put out an open request and he approached me
Other (please say why ____________)

LOOP Q14i FOR FURTHER COUPON RECIPIENTS
[IF Q14>1] Concerning the second person you gave a coupon to:
[IF Q14>2] Concerning the third person you gave a coupon to:

TRANSFORM Ithuluzi Lohlolo

Ilanga Lokuhlolwa: [unyaka/Inyanga/usuku]

Igama lomhloli: ___________________

Inombolo yepasi kambambiqhaza: ______________________

Siyabonga ukuvuma ukwenza lolu hlolo. Manje sizokubuza imibuzo emayelana nawe. Sicela ukhumbule ukuthi akumele uphendule imibuzo ongafuni ukuyiphendula futhi uyakwazi ukumisa lolu hlolo noma ingasiphi isikhathi.

IMIBUZO YOKUZIJWAYEZA

Kunezinhlobo ezintathu zemibuzo eyinhloko, ejwayeleke kakhulu inamabhathini ayindilinga eceleni kwempendulo, futhi ikuvumela ukuthi ukhethe impendulo eyodwa. Uma usyichofozile impendulo, umbuzo uzonyamalala bese izoveza umbuzo olandelayo. Uma wenze iphutha, chofoza ibhathini eliwumcibisholo elikhomba esinxeleni ("EMUVA"), elingezansi kweskrini, esinxeleni. Awukuzame lokhu.

Ngabe ubukeka kahle kangakanani?

Ngibukeka kahle
Ngibukeka kahle kakhulu
Ngibukeka kahle okungakholakali

Eminye imibuzo inamabhathini amancane AYIZIKWELE eceleni kwezimpendulo futhi ungachofoza noma ezingaki izimpendulo ozithandayo. Uma usuyikhethile impendulo umbuzo ngeke inyamalale-uma usuqedile kudingakala ukuthi uchofoze ibhathini eliwumcibisholo okhomba kwesokudla ("OKULANDELAYO") elingezansi kwesokudla. Uma wenze iphutha, chofoza ibhathini eliwumcibisholo elikhomba esinxeleni ("EMUVA"), elingezansi, esinxeleni. Awukuzame lokhu.

Ngabe iziphi izinhlobo zemidlalo ozibuka kwiTV kulezi ezilandelayo?
Isaziso lemibuzo ijwayele ukuba nesinezezelo esibhalwe ukuthi "OKUNYE"-uma uchofoza lokhu, kudingeka ukuthi uyibhale lenye impendulo yakho. Chofoza "OKUNYE" mase ubhale "Isibhakela" (noma omunye umudlalo owubukayo).

Ibhola
IRugby
ICricket
Okunye-sicela ukusho: (Kubhale)

Eminye imibuzo embalwa ibuza ukuthi ugcine nini ukwenza into ethize, bese ikuvezela ikhalenda ukuze ukhethe usuku, inyanga Kanye nonyaka.Uma lokhu kwenzeka uyakwazi ukudlulisa izinyanga neminyaka usebenzisa imicibisholo ekhoma esandleni sesinxele noma kwesokudla kuyona ikhalenda. Uma usulukhethile usuku oluyimpendulo yakho, chofoza u "Set" ukukugcina okukhethile. Uma usuchofoze u "Set" umbuzo uzonyamalala bese kuvela olandelayo. Awukuzame lokhu.

Ugcine nini ukukhuluma noyedwa kumalunga omndeni wakho?

Kehetha usuku (usuku, inyanga, unyaka)

AX. Coupon management

Manje sesizoqala iseveyi kahle- ngicela uphendule yonke imibuzo ngeqiniso. Ngicela ukhumbule ukuthi asikho isidingo sokuthi uphendule imibuzo ongathandi ukuyiphendula futhi ungayimisa iseveyi nganoma ingasiphi isikhathi. Zonke izimpendulo zakho ziyimfihlo ngokuphelele.

Uhlu lwemibuzo yokuqala ingokuthi, wazi kanjani ngalolucwaningo, nokuthi uyithathephi ikhuphoni.

AX1. Umazi kanjani lomuntu okuphe ikhuphoni ukuthi ubambe iqhaza kukelithuluzi lohlolo?

Umngani osendelene nami

Umngani

Umuntu engizwana naye

Umuntu engingamazi

Okunye

Q1a, Uma u Q1 = Okunye buza, Ngabe umazi ngayiphi enye indlela?

[Bhala]

AX2. Bewulaphi ngesikhathiwuthola lelikhuphoni lokuzobamba iqhaza kulolucwaningo?

Ekhaya noma eduzane nasekhaya

Emsebenzini noma eduzane nasemsebenzini

Siphumile emgwaqeni

Endaweni zokuphuza nokuzijabulisa

Ngaphandlekwalelihovisi

Kwenye (Isho laphi ____________)

AX3. Ukube wawubambe iqhaza kuqala kulesaveyi, ucabanga ukuthi bekungenzeka umnikeze ikhuphoni lomuntu okunikezile?

Yebo

Cha

AX4. Ngaphandle kwalomuntu okunikeze lelikhuphoni ofikenayo namhlanje, ngabe ukhona omunye umuntu okewazama ukukunikeza ikhuphoni?

Yebo

Cha

AX5. Uma uvuma, lokhu kwenzeke kangaki?

[____] izikhathi

A. Imibuzo eqondene nawe (1).

Uhlu lokuqala lwemibuzo lungawe, iminyaka yakho, lapho ohlala khona, Kanye nalapho owazalelwa khona. Leseveyi isiqalile-zonke izimpendulo zakho ziyimfihlo. Sicela uphendule imibuzo ngeqiniso.

A1. Uneminyaka emingaki?

______ (Iminyaka)

A2. Ingabe uhlala kuyiphi indawo?

[SHOW IF SITENAME= NAIROBI]

Dagoretti
Starehe

Embakasi
Kasarani
Langata
Makadara
Kamukunji
Westlands
Other (Ngicela ucacise) _____________

SHOW IF SITENAME[SHOW IF SITENAME= SOUTH AFRICA]

[SHOW IF SITENAME = Johannesburg SOUTH AFRICA]

Braamfontein
Diepkloof,
Hillbrow
Orange Farm
Rosebank
Rosetonville
Sandton,
Soweto
Yeoville
Other (please specify) _____________

A3. Ngabe wazalelwa e [Kenya / South Africa]?

Cha
Yebo **[iya ku A5]**

A4. [uma u A3 kuwu Cha] Ingabe wazalelwa kuliphi izwe eAfrika?

Yebo
Cha

A4a. [If A4 is yes] Iliphi izwe owazalelwa kulo eAfrika?

[Khetha kulohlu lwamazwe]

A5. Ingabe wazalelwa e [SITE NAME]?

Cha

Yebo

A6. [If A5 is no] Iliphi idolobha owazalelwa kulo? e?

Idolobha/idolojana___________

A7. Ingabe usuhlale iminyaka emingaki e[SITE NAME]??

A. Imibuzo eqondene nawe (2).

A8. Ingabe wagcina kuliphi ibanga eskolweni owaliqeda?

[SHOW IF SOUTH AFRICA]

Angifundanga

Amabanga okuqalisa

Amabanga aphakeme

Amabanga alekayo noma eTechnical Secondary

Ikolishi noma inyuvesi noma isikhungo esiphakeme semfundo

[SHOW IF KENYA]

Angifundanga

Amabanga okuqalisa

Amabanga aphakeme

Amabanga alekayo noma eTechnical Secondary

Ikolishi noma inyuvesi noma isikhungo esiphakeme semfundo

A9. Iyiphi indlela echaza isimo sakho sokusebenza samanje?

Ngiqhashwe ngokwesikhathi esiphelele
Ngiqhashwe ngesikhathi esingaphelelanga
Ngiyazisebeza
Angisebenzi noma ngiphakathi kwemisebenzi
Okunye (__________)

A11. Ngabe wenzemalini kulenyanga edlule?

[SHOW IF KENYA]

KSH [faka inani lemali]

[SHOW IF SOUTH AFRICA]

ZAR [faka inani lemali]

A12. Kkufaka wena phakathi, bangaki abantu ababheke lomholo?

[Faka inani]

A. Imibuzo eqondene nawe (3).

A13. Ngabe eyiphi inkolo oyilandelayo?

Amakrestu
Islam
ubuHindu
okunye
Anginayo engiyilandelayo

A14. Ngabe unguluphi uhlanga?

Ngiwumuntomnyama
Ikhaladi
Indiya/ningowase Asia
Ngiwumlungu
Ngincama ukungasho

Okunye

A15. [SOUTH AFRICA ONLY] Ingabe ubuchaza kanjani ubuzocansi bakho?

Ngiyindoda eya ecansini namanye amadoda.

Ngiyindoda eya ecansini nabantu besifazane futhi namanye amadoda.

Ngiyindoda eya ecansini nabafazi kuphela.

Okunye (usungachaza)________________

Angizazi

A16. [SHOW IF KENYA]. Ubuchaza kanjani ubusocansi bakho ngesiSwahili?

Basha
Hanithi
Kuchu
Kuruzi
Msago
Msenge
Shoga
Okunye (shano ukuthi kuphi______________)

A16. Yibuphi ubulili bakho bokuzalwa, (isibonelo, obuvela esitifiketini sokuzalwa)?

Ngingowesilisa

Ngingowesifazan

Angifuni ukuphendula lomubuzo

A17. Ngabe ubulili bakho ubuchaza kanjani?

Owesilisa

Owesifazane

Ubulili obushintshiwe

Angiyena owesilisa, owesifazane noma obulili obushintshiwe

A18. Ngabe sithini isimo sakho sokushada?

Ngishadile/ umshado wenkantolo/ sihlalisene ngokusemthethweni
Ngisingili/ngihlukanisile/ ngiwumfelokazi**[SKIP TO NEXT SECTION]**

A19. Ngabe ubulili buni uphathina wakho?

Owesilisa

Owesifazane

Ubulili obushintshiwe

Okunye

B. EZENHLALO: ABANGANI

Manje sizokubuza imibuzo ngobungani bakho emadodeni aya ocansini namanye amadoda.

B1. Ingabe mangaki amadoda aya ecansini namanye amadoda owaziyo oke waxoxisana nawo kulenyanga edlule?

Ngokuthi 'owaziyo' sisho ukuthi umuntu enazana naye anamagama enixoxisene nabo, kungaba nibonene, noma ngocingo, nithumelane imilayezo ngocingo noma online?

[faka inani]

B2. Ingabe mangaki kulamadoda [B5] azanayo?

bonke noma abaningi bayazana
Abaningi babo bayazana [around <ROUND[B5*.75]> of them]
Uhhafu wabo bayazana [around <ROUND[B5*.5]> of them]
Abanye, kodwa hhayi abaningi bayazana [around <ROUND[B5*.25]> of them]
Abancane kakhulu abazanayo

B3 Ingabe mangaki lamadoda[B1] osuke wahlangana nawo?

[faka inani]

B4. . ingabe mangaki kulamadoda [B3] abangaphezu kweminyaka ewu 18 noma ngaphezulu?

[faka inani]

B5.Ingabe mangaki kulamadoda [B4] abahlala edolobheni elifanayo nawe?

[faka inani]

B6Ingabe mangaki kulamadoda [B5] oke wababona kulamaviki amabili adlule?

[faka inani]

.

B7. Ingabe mangaki kulamadoda [B5] abangaphezu kweminyaka ewu 30?

[faka inani]

Loluhlu lwemibuzo elandelayo ibuza ngezindlela ohlangana nazo namanye amadoda aya ocansini namanye amadoda.

B8. Ingabe wagcina nini ukuvakashela lezindawo ukubonana namadoda aya ocansini namanye amadoda?

	Ngivakashile ngenyanga edlule	Ngivakashile ngonyaka odlule kodwa hayi enyangeni edlule	Ngivakashile kudlula unyaka.	Angikaze ngivakashe ngalesizathu
Umhlangano wangaphandle isb. epaki, emgaqweni noma ebhishi	◯	◯	◯	◯
EBar noma eklabhini	◯	◯	◯	◯
Endlini yomngani noma eyami.	◯	◯	◯	◯
ESauna, eBathhouse noma ephathini yocansi	◯	◯	◯	◯
Endaweni yomphakathi yamadoda alala namanye amadoda isb. iDrop in Centre noma isikhungo samadoda alala namanye amadoda	◯	◯	◯	◯

B9. Ugcine nini ukusebenzisa iwebhusayithi noma ama ephu ukwakha ubungani namanye ama MSM?

Angikaze [jump to B12]
Kulenyanga edlule
Kulonyaka odlule
Ngaphezu konyaka manje

B10. Ngabe yimaphi kulamasevisi einthanethi osoke wawasebenzisa ukwakha ubungani nama MSM kulenyanga edlule [maka ibhokisi elinempendulo/izimpendulo zakho?

2go ◯
Adam4Adam ◯
Badoo ◯
Facebook ◯
Gay Radar ◯
Gay.com ◯
Gaydar ◯
Gayxchange ◯
Hornet ◯
Instagram ◯
iPlay ◯
Mambaonline ◯
Radar ◯
Red Velvet ◯
Scruff ◯
Sex Trader South Africa ◯
Skype ◯
Snapchat ◯

		Get Male	◯	Manhunt	◯		
		Grindr	◯			Twitter	◯
				ManToManPlus	◯	Twoo	◯
Dating Buzz	◯	Guy Spy	◯	Men2Men	◯	WeChat	◯
						Whatsapp	◯
				MISTER	◯	Amanye ________	
		Hookups	◯	Planet Romeo	◯		

B11. [Ngo Janwari 1 kulonyaka], ingabe ububhalisele ukusebenzisa lamasevisi elandelayo?

[SHOW IF COUNTRY=ALL]

B11.a. Grindr

Yebo
Cha

B11.b. Planet Romeo

Yebo
Cha

B11.c. Hornet

Yebo
Cha

[SHOW IF COUNTRY=SOUTH AFRICA]

B11.d. Mamba Online

Yebo
Cha

[If yes to B11a] B11.e. NgoJanwari 1 kulonyaka, ubunamaprofayela amangaki kuGrindr?

[Faka inani]

[If yes to B11b] B11.f. NgoJanwari 1 kulonyaka, ubunamaprofayela amangaki kuPlanet Romeo?

[Faka inani]

[If yes to B11c] B11.f. NgoJanwari 1 kulonyaka, ubunamaprofayela amangaki kuHornet?

[Enter number]

[If yes to B11d] B11.g. NgoJanwari 1 kulonyaka, ubunamaprofayela amangaki kuMamba Online?

[Enter number]

B12. Uke wavakashela noma wamukela imininingwane kunhlangano yomphakathi noma iqembu lokwesekana lamaMSM?

Yebo
Cha [JUMP TO NEXT SECTION]
Angazi/anginasiqiniseko [JUMP TO NEXT SECTION]

B13. Iyiphi kulezizinhlangano ezeseka abantu abathandana nabanye bobulili obufanayo oke wazivakashela kulonnyaka odlule?

[SHOW IF COUNTRY=SOUTH AFRICA]

SOHACA
(ANOVA)Health4Men
LGBTI
ACTIVATE
GALA
Other …

[SHOW IF COUNTRY=KENYA]

ISHTAR
GALCK
HOYMAS

]

C. UKWESEKWA KWENHLALO

Loluhlu lwemibuzo elandelayo angokwesekwa ngokwenhlalo- noma ukwesekwa okuthola kuphathina/ophathina, abangani Kanye nomndeni.

Ngicela usho ukuthi uvuma noma awuvumi kangakanani kulezitatamende ezilandelayo

	Angivumi nhlobo	Angivumi kakhulu	Angivumi nje	Ngiphakathi nendawo	Ngivuma kancane	Ngiyavuma nje	Ngivuma kakhulu
**C1. Kukhona umuntu obalulekile kimina ohlezi akhona mangimudinga**							
	○	○	○	○	○	○	○
**C2. Kukhona umuntu obalulekile kimina ohlale akhona ezikhathini zobumnandi nobubi**							
	○	○	○	○	○	○	○
**C3. Umndeni wami uyazama ukungisiza**							
	○	○	○	○	○	○	○
**C4. Umndeni wami uyangeseka ngokomoya**							
	○	○	○	○	○	○	○
**C5. Kukhona umuntu ongumduduzi empilweni yami**							
	○	○	○	○	○	○	○
**C6. Abangani bami bangeseka ngaso sonke isikhathi**							
	○	○	○	○	○	○	○
**C7. Abangani bami bahlale bengisekile ima izinto zingahambi kahle**							
	○	○	○	○	○	○	○
**C8. Ngiyakhona ukukhuluma ngezinkinga zami nomndeni wami**							
	○	○	○	○	○	○	○
**C9. Nginabangani esihlale sesekana nabo ngezikhathi ezimbi nezikhathi ezinhle**							
	○	○	○	○	○	○	○
**C10. Nginomuntu empilweni yami okhathalela imizwa yami**							
	○	○	○	○	○	○	○
**C11. Umndeni wami uyanginceda ekuthatheni izincumo ezibalulekile**							
	○	○	○	○	○	○	○
**C12. Ngiyakhona ukukhuluma nabangani bami ngezinkinga engihlangabezana nazo**							

	○	○	○	○	○	○	○

D. EZENHLALO: UKWECWASANA NOBANDLULULO NGOKWEZOBULILI

Loluhlu lwemibuzo elandelayo ingokwazisa abantu ngokuthi uyindoda eyaocansini namanye amadoda kanye nezinkinga ezibangwe ilokhu.

Ngicela uqaphele mawuphendula lemibuzo. Eminye ikucela ukuthi ucabange ngezinyanga eziwu 12 ezedlule, ezinye zikucela ukuthi ucabange ngempilo yakho yonke.

D1. Uke wezwa ngathi emndenini wakho bayakukhipha emisebenzini yasekhaya ngoba uyindoda eya ecansini namanye amadoda?

Yebo

Cha

D2. Uke wezwa ngathi amalunga omndeni wakho ayakubandlulula noma bahleba ngawe ngoba uyindoda eya ecansini namanye amadoda.

Yebo

Cha

D3. Jikelele, kunzima kangakanani ukufihlela UMNDENI wakho ngokulala namanye amadoda?

Ngizama kakhulu ukukufihla
Ngiyazama nje ukukufihla
Angikuifihli, kodwa angithandi ukukhuluma ngakho
Ngiyakhuluma ngakho
Akungeni

D4. Jikelele, uzama kangakanani ukufihlela abangani bakho ukuthi uya ocansini namanye amadoda?

Ngizama kakhulu ukukufihla
Ngiyazama nje ukukufihla
Angikuifihli, kodwai angithandi ukukhuluma ngakhoo
Ngiyakhuluma ngakho
Akungeni

D5. Uke wezwa engathi abangani bakho abathandi ukuzibandakanya nawe ngenxa yokuthi uya ocansini namanye amadoda?

Yebo

Cha

D6. Jikelele, uzama kangakanani ukugcina kuyimfihlo kubasebenzi basemitholampilo ukuthi uyindoda eya ocansini namanye amadoda?

Ngizama kakhulu ukukufihla
Ngiyazama nje ukukufihla
Angikuifihli, kodwai angithandi ukukhuluma ngakhoo
Ngiyakhuluma ngakho
Akungeni

D7. Kulezinyanga eziwu 12 ezedlule, uke wezwa usaba ukuya emtholampilo ngoba usaba ukuthi kukhona umuntu ongathola ukuthi uyindoda eya ocansini namanye amadoda?

Yebo
Cha

D8. Kulezinyanga eziwu 12 ezedlule, uke wezwa ungaphathekanga kahle emitholampilo ngoba kukhona umuntu owaziyo ukuthi uyindoda eya ocansini namanye amadoda?

Yebo
Cha

D9. Kulezinyanga eziwu 12 ezedlule, uke wezwa uhletshwa noma uhlekwa abasebenzi basemtholampilo ngoba uya ocansini namanye amadoda?

Yebo
Cha

D10. Kulezinyanga eziwu 12 ezedlule ukwe wezwa ukuthi amaphoyisa awafuni ukukusiza ngoba uya ocansini namanye amadoda?

Yebo
Cha

D11. men Kulezinyanga eziwu 12 ezedlule uke wezwa usaba ukuhamba emphakathini ngoba uya ocansini namanye amadoda?

Yebo
Cha

D12. Kulezinyanga eziwu 12 ezedlule, ukewacelwa ivalamlomo ngenxa yokuthi uya ocansini namanye amadoda

Yebo

Cha

D13. Kulezinyanga eziwu 12 ezedlule, kukhona umuntu okulimeze ngokomzimba (akuphushe, akuqindezele ekhoneni, akushaye ngempama, akushaye, akukhame, noma akulimaze ngokomzimba)?

Yebo

Cha **[JUMP TO D20]**

D14. Ubani owenze lokhu? [Maka konke okufunayo]

Umuntu engingamazi

Ilunga lomndeni

Iphoyisa noma isisebenzi somphakathi esisemthethweni

Umtholisi mpilo

Uphathina

Obethenga ucansi

Okunye

D15. Kulezinyanga eziwu 12 ezedlule, uke waphoqwa ukuthi uye ocansini ungafuni? (ngokuphoqa sichaza ukuthi Uphocwe ngokomzimba, ubanjwe ukuthi wenze ucansi, noma bakufake izinto, ungafuni).

Yebo

Cha [**JUMP TO D24**]

D16. Ubani owenze lokhu? [Maka konke okufunayo]

Umuntu engingamazi

Ilunga lomndeni

Iphoyisa noma isisebenzi somphakathi esisemthethweni

Umtholisi mpilo

Uphathina

Obethenga ucansi

Okunye

D17. Kulezinyanga eziwu12 ezedlule, uke wezwa usatshiswa ngokuthi uzobanjwa noma uzoboshwa yiphoyisa ngenxa yokuthi uyaocansini namanye amadoda?

Yebo

Cha

D18. Uke waboshwa ngenxa yokuthi uyaocansini namanye amadoda?

Yebo

Cha

ngesikhathi

E. UKUZIPHATHA NGEZOCANSI

Singathanda mnje ukukubuza ngendlela yokuziphatha ngokwezocansi okusandakwenzeka. Ngocansi sichaza nama ikuphi okuphathelene nokuthinta izitho zangasese- sibala indlwabu (okwesitho sangaphambili, nokuzithokozisa ngomunwe esithweni sangasese); ucansi olwenziwa ngomlomo (ukumunca/iblowjob,ukukhotha, ukukhotha isitho sangasese sangemuva); ucansi lwesithosangasese sangaphambili sowesifazane nome lwesitho sangemuva.

E1. Kulezinyanga eziwu 3 ezedlule, ngabe ulwenzile ucansi nomuntu wesilisa?

Yebo

Cha

E1a. [If E1 = yes] be mangaki amadoda ahlukene oye nawo ocansini kulezizinyanga eziwu 3 ezedlule? [Faka inani]

E2. Kulezinyanga eziwu 3 ezedlule ngabe uluphi locansi lwesitho sangasese sangemuva oke walenza?

Ububhothomu kuphela

Ubuthophu kuphela

Ubuvesethayela

Akukho kuloku okubaliwe

E3 [if E2= receptive or both] Kulezinyanga eziwu 3 ezedlule, ngabe uwasebenzise kangakanani amakhondomu ngesikhathi wenza ucansi futhi uyi bhothomu?

Njalo

Ngesikhathi esiningi

Ngesinye isikhathi

Akukavami

Akwenzeki

E4. [if E2= receptive or both] Kulezinyanga eziwu 3 ezedlule, ngabe uwasebenzise kangakanani amakhondomu ngesikhathi uyi thophu?

Njalo

Ngesikhathi esiningi

Ngesinye isikhathi

Akukavami

Akwenzeki

E5. Kulezinyanga eziwu 3 ezedlule ngabe wenze hlobolupi locansi lomlomo nabantu besilisa?

Ngimuphe iblow job

Ungiphe iblow job

Ngmuphile futhi ngaphiwa iblowjob

Akukho ngenhla

E6. Kulezinyanga eziwu 12 ezedlule ngabe kukhona amadoda akukhokhele ukuthi wenze ucansi? Ngokukhokhelwa sisho imali, izipho noma ukusizwa ukuze wena wenze ucansi nabo.

Yebo

Cha

E7.. Kulezinyanga eziwu 12 ezedlule, ngabe uke wakhokhela indoda ukuthi iye ocansini nawe? Ngokukhokhelwa sisho imali, izipho noma uyisize ukuze yona yenze ucansi nawe. Yebo

Cha

E8. Kulezinyanga eziwu 12 ezedlule ingabe ukhona kophathina bakho oke wazama ukukulimaza? Ngaloku shisho ukukuphusha, akucindezele phansi, akushaye ngenqindi, akukhahlele, azame ukukuklinya, akuhlasele ngommese, ngesibhamu noma ngesinye isikhali.

Yebo

Cha

E9. Kulezinyanga eziwu 12 ezedlule, ngabe uphathina wakho wendoda usebenzise udlame noma wakuthusa ngomlomo ukuze uye ocansini naye wena ungafuni?

Yebo

Cha

E10. Kulezinyanga eziwu 3 ezedlule uke walwenza ucansi nomuntu wesifazanei?

Yebo

Cha

E10a. Ngabe bangaki abantu besifazane owenze ucansi nabo kulezinyaka eziwu3?

[Faka inani]

E11. Kulezinyanga eziwu 3 ezedlule ingabe uluphi uhlobo locansi oke walwenza nabesifazane?

Ucansi lwesitho sangasese sabesifazane sangaphambili

Ucansi lwesitho sangasese sangemuva

Kokubili

Akukho

E12. [if E11= vaginal or both] **Kulezinyanga eziwu 3 ezedlule uyisebenzise kangakanani ikhondomu ngesikhathi wenza ucansi lwesitho sangansense sabesifazane sangaphambili nophathina wesifazane?**

Njalo

Ngesikhathi esiningi

Ngesinye isikhathi

Akukavami

Akwenzeki

E13 [if E11=anal or both] **Kulezinyanga eziwu 3 ezedlule usebenzise kangakanani amakhondomu mawenza ucansi lwesitho sangasese sangemuva nomuntu wesifazane?**

Njalo

Ngesikhathi esiningi

Ngesinye isikhathi

Akukavami

Akwenzeki

E14. Kulezinyanga eziwu 12 ezedlule ngabe ukhona owesifazane okukhokhele ukuthi uye naye ocansini? Ngokukhokhelwa sisho ukuthi akuphe imali noma izipho noma akusise ngokuthi ufuna ucansi.

Yebo
Cha

E15. Kulezinyanga eziwu 12 ezedlule, ngabe uke wakhokhela owesifazane ukuthi aye ocansini nawe? Ngokukhokhela sisho ukuthi akuphe imali noma izipho noma akusise ngokuthi ufuna ucansi.

Yebo
Cha

EX. UKUZIPHATHA KWEZOCANSI

Manje sizokubuza imibuzo ngabantu ABANE oye nabo ocansini phambilini. Khetha igama lokuteketisa nima izicu zamagama abo kuze ukwazi ukubahlukanisa. Akumelanga kuba amagama abo angempela, kumele kube amagama nje ozokhona ukubahlukanisa ngawo.

Khumbula masikhuluma ngocansi, sibala indlwabu (okwesitho sangaphambili, nokuzithokozisa ngomunwe esithweni sangasese); ucansi olwenziwa ngomlomo (ukumunca/iblowjob,ukukhotha, ukukhotha isitho sangasese sangemuva); ucansi lwesithosangasese sangaphambili sowesifazane nome lwesitho sangemuva.

Uphathina #1:
Ubani ogcine ukuya naye ocansini? (Write in a nickname, first name or initials)
[Faka amanishela]

Uphathina # 2:
Ngaphambi kuka (Partner #1) Ubani ogcine ukuya naye ocansini? (Bhala igama lokudlala, Elokuqala noma amanishela) [Faka amanishela]

Uphathina #3:
Ngaphambi kuka (Partner #2) Ubani ogcine ukuya naye ocansini?? (Bhala igama lokudlala, Elokuqala noma amanishela) [Faka amanishela]

Uphathina #4:
Ngaphambi kuka (Partner #3) Ubani ogcine ukuya naye ocansini? (Bhala igama lokudlala, Elokuqala noma amanishela) [Faka amanishela]

ROUTE 1: A CASUAL, ONE-OFF SEXUAL PARTNER
[Partner #1]

Ngabe eliphi ilanga [eliduze kunanamuhla] ogcine ngalo ukwenza ucansi no[Partner #1]. Qagela uma ungasakhumbuli kahle

[faka ilanga]

U [Partner #1] ubulili buni?

Owesilisa

Owesifazane

Obulili bushintshile

angazi

Wayeneminyaka emingaki u[Partner #1] ngesikhathi nigcina ukwenza ucansi? Qagela uma unganasiqiniseko. [Faka iminyaka]

Nahlangana kanjani okokuqala no [partner #1]?

ngabangani

ngomndeni

eskolweni

emsebenzini

endaweni yokuzijabulisa

emcimbini

Kwi social network-isho ukuthi yiphi

Okunye-isho ukuthi laphi

Angikhumbuli

Ngabe ucansi usulwenze kangaki no [partner #1]

Kanye kuphela

Kaningana

Ngabe ucabanga ukuthi usazophinde wenze ucansi no [partner #1] futhi?

Yebo

Cha

Ngabe wamunika u**[Partner #1] imali, izipho noma wamusiza ukuze enze ucansi nawe?**

Cha

Yebo

Ngabe u**[Partner #1]** wayekunika **imali, izipho noma wakusiza ukuze wenze ucansi naye?**

Cha

Yebo

EX28 [i] [if EX3=male] Ngabe nanenza luphi uhlobo locansi [Partner #1]? [Bheka okungenayo]

Ngangiyi Bhothomu

Ngangiyi Thophu

Ngamupha iblow job
Ngaphiwa iblow job

EX29A[i] [if EX3=male & EX28 = receptive anal sex] Isikhathi ngangiyibhothomu no [Partner #1], Ngabe nasebenzisa ikhondomu noma cha?

Ngekhondomu
Ngaphandle kwekhondomu

EX29B[i] [if EX3=male & EX28 = insertive anal sex] Mawugcina ukuba yiThophu no [Partner #1], loku kwakunge noma ngaphandle kwekhondomu?

Ngekhondomu
Ngaphandle kwekhondomu

EX30[i] [if EX3=female] uhlobo luni locansi enalwenza no[Partner #1]? [Khetha okuyikona]

ucansi lwesitho sangasese sabesifazane sangaphambili
Ucansi lwesitho sangasese sangemuva
Ucansi lomlomo

EX31A[i] [if EX3= female & EX28 = vaginal sex] ngesikhathi wenza ucansi lwesitho sangasese sabesifazane sangaphambili no [Partner #1], loku kwakunge noma ngaphandle kwekhondomu?

Ngekhondomu
Ngaphandle kwekhondomu

EX31B[i] [if EX3= female & EX28 = anal sex] ngesikhathi nigcina ukwenza ucansi lwesitho sangasese sangemuva no [Partner #1], loku kwakunge noma ngaphandle kwekhondomu?

Ngekhondomu
Ngaphandle kwekhondomu

EX33 [i] Uke u [Partner #1] wakutshela ngesimo sakhe seHIV?

Yebo
Cha **[JUMP TO E35 [i]]**
Angazi

EX34 [i] Sithini isimo sika [Partner #1] seHIV?

Une HIV
Akanayo iHIV
Angazi

SKIP TO E36[i]

EX35[i] Wawukholwa ukuthi isimo sika [Partner #1] seHIV sithini ngesikhathi nenza ucansi naye?

Ngicabanga ukuthi **[Partner #1]** angaba nayi iHIV
Ngicabanga ukuthi u **[Partner #1]** akanayo iHIV

Angazi

ROUTE 2: A REGULAR SEXUAL PARTNER WHO THEY EXPECT TO HAVE SEX WITH AGAIN IN THE FUTURE

Ngabe eliphi ilanga [eliduze kunanamuhla] ogcine ngalo ukwenza ucansi no[Partner #1]. Qagela uma ungasakhumbuli kahle

[Faka ilanga]

U[Partner #1] ubulili buni?

Owesilisa
Owesifazane
Ubulili obushintshile
Angazi

Wayeneminyaka emingaki u[Partner #1] ngesikhathi nigcina ukwenza ucansi? Qagela uma unganasiqiniseko.

[Faka iminyaka]

Nahlangana kanjani okokuqala no [partner #1]?

Ngabangani
Ngomndeni
Eskolweni
Emsebenzini
Endaweni yokuzijabulisa
Emcimbini
Kwi social network-isho ukuthi yiphi
Okunye-isho ukuthi laphi
Angikhumbuli

Ngabe ucansi usulwenze kangaki no [partner #1]

Kanye kuphela
Kaningana

Ngabe ucabanga ukuthi usazophinde wenze ucansi no [partner #1] futhi?

Yebo
Cha

EX20A[i] [if EX19i=YES] Yikuphi kulokhu okulandelayo okuchaza ubudlelwane bakho no [Partner #1] bamanje?

Sishadile
Sesibe izithandani isikhathi eside
Singabangani ababuye benze ucansi
Sihlanganiswa ucansi

EX21A[i] [if EX19i=YES] Uhlala naye u [Partner #1]?

Yebo
Cha

EX22A[i] [if EX19i=YES] Uyamthanda u [Partner #1]?

Yebo, kakhulu
Yebo, kancane
Cha

EX24A[i] [if EX19i=YES] Ngabe ubheke ku <EX1[i]> ukuthola imali, umholo noma indawo yokuhlala?

Yebo, kakhulu
Yebo, kancane
Cha

EX26[i]. Uke wamupha u [Partner #1] imali, izipho noma usizo, ubheke ukuthola ucansi?

Yebo
Cha

EX27[i]. Uke u [Partner #1] akuphe imali, izipho noma usizo, ebebheke ukuthola ucansi?

Yebo
Cha

EX28[i] [if EX3=male] Ngabe nanenza luphi uhlobo locansi no[Partner #1] ? [bheka okungenayo]

Ngangiyi Bhothomu
Ngangiyi Thophu
Ngamupha iblow job
Ngaphiwa iblow job

EX29A[i] [if EX3=male & EX28 = receptive anal sex] Ngesikhathi uyibhothomu no [Partner #1], Ngabe nayisebenzisa ikhondomu noma cha?

Ngekhondomu
Ngaphandle kwekhondomu

EX29B[i] [if EX3=male & EX28 = insertive anal sex] Mawugcina ukuba yoThophu no [Partner #1] loku kwakunge, noma ngaphandle kwekhondomu?

Ngekhondomu
Ngaphandle kwekhondomu

EX30[i] [if EX3=female] uhlobo luni locansi enalwenza no<EX1[i]>? [Khetha okuyikhonaokuyikona]

Ucansi lwesitho sangasese sowesifazane sangaphambili
Ucansi lwesitho sangasese sangemuva
Ucansi lomlomo

EX31A[i] [if EX3= female & EX28 = vaginal sex]
Ngesikhathi nigcina ukwenza ucansi lwesitho sangasese sangaphambili sowesifazane no [Partner #1], ngabe lokhu kwakunge, noma ngaphandle kwekhondomu?

Ngekhondomu
Ngaphandle kwekhondomu

EX31B[i] [if EX3= female & EX28 = anal sex]

Ngesikhathi nigcina ukwenza ucansi lwesitho sangasese sangemuva no[Partner #1], ngabe lokhu kwakunge, noma ngaphandle kwekhondomu

Ngekhondomu

Ngaphandle kwekhondomu

EX33[i] Uke u [Partner #1] wakutshela ngesimo sakhe seHIV?

Cha [JUMP TO E35[i]]

Yebo

Angazi

EX34[i] Sithini isimo sika [Partner #1] seHIV?

Une HIV

Akanayo iHIV

Angazi

SKIP TO E36[i]

EX35[i] Ukholwa ukuthi isimo sika [Partner #1] seHIV sithini?

Ngicabanga ukuthi angaba nayo iHIV

Ngicabanga ukuthi akanayo iHIV

Angazi

EX36[i] Uke wena no [Partner #1] nakhaphana ukuyowenza loku okulandelayo [khetha okuyikho]

Ukuyolulekwa nokuhlola iHIV

Kuphoyinti lasemtholampilo wezocansi

Kuphoyinti lomtholampilo wokulapha iHIV

Umhlangano wezempilo wabantu besilisa abaya ocansini nabanye abantu besilisa

Umcimbi ohlelewe inhlangano yabantu besilisa abaya ocansini nabanye besilisa

Cha, akukho kulokhu okungenhla

ROUTE 3: A FORMERLY REGULAR SEXUAL PARTNER WHO THEY DO NOT EXPECT TO HAVE SEX WITH AGAIN IN THE FUTURE

Ngabe eliphi ilanga [eliduze kunanamuhla] ogcine ngalo ukwenza ucansi no[Partner #1]. Sicela uagele uma ungasakhumbuli kahle

[Faka ilanga]

U[Partner #1] ubulili buni?

Owesilisa

Owesifazane

Ubulili obushintshile
Angazi

Wayeneminyaka emingaki u[Partner #1] ngesikhathi nigcina ukwenza ucansi? Sicela uagele uma unganasiqiniseko.
[Faka iminyaka]

Nahlangana kanjani okokuqala no [partner #1]?
Ngabangani
Ngomndeni
Eskolweni
Emsebenzini
Endaweni yokuzijabulisa
Emcimbini
Kwi social network-isho ukuthi yiphi
Okunye-isho ukuthi laphi
Angikhumbuli

Ngabe ucansi usulwenze kangaki no [partner #1]
Kanye kuphela
Kaningana

Ngabe ucabanga ukuthi usazophinde wenze ucansi no [partner #1] futhi?
Yebo
Cha

EX20B[i] [if EX19i=YES] Yikuphi kulokhu okulandelayo okuchaza ubudlelwane owawunabo no [Partner #1] bamanje?
Sasishadile
Sesiyizithandani isikhathi eside/ophathina abaqavile
Sasingabangani ababebuye benze ucansi
Sasihlanganiswa ucansi

EX21B[i] [if EX19i=NO & EX18i=YES] Nisenobudlelwano, wawuhlala naye u <EX1[i]>?
Yebo
Cha

EX22B[i] [if EX19i=NO & EX18i=YES] Nisenobudlelwano, wawumthanda u <EX1[i]>
Yebo, kakhulu
Yebo, kancane
Cha

EX24B[i] [if EX19i=NO] Ngabe wawubheke ku <EX1[i]> ukuthola imali, umholo noma indawo yokuhlala?
Yebo, kakhulu
Yebo, kancane
Cha

EX26[i]. Uke wamupha u [Partner #1] imali, izipho noma usizo, ubheke ukuthola ucansi?

Yebo

Cha

EX27[i]. Uke u [Partner #1] wakupha imali, izipho noma usizo, ebebheke ukuthola ucansi?

Yebo

Cha

EX28[i] [if EX3=male] Ngabe nanenza luphi uhlobo locansi no[Partner #1] ? [bheka okungenayo]

Ngangiyi Bhothomu

Ngangiyi Thophu

Ngamupha iblow job

Ngaphiwa iblow job

EX29A[i] [if EX3=male & EX28 = receptive anal sex] Ngesikhathi uyibhothomu no [Partner #1], Ngabe nayisebenzisa ikhondomu noma cha?

Ngekhondomu

Ngaphandle kwekhondomu

EX29B[i] [if EX3=male & EX28 = insertive anal sex] Mawugcina ukuba yoThophu no [Partner #1] loku kwakunge, noma ngaphandle kwekhondomu?

Ngekhondomu

Ngaphandle kwekhondomu

EX30[i] [if EX3=female] uhlobo luni locansi enalwenza no[Partner #1]? [Khetha okuyikhonaokuyikona]

Ucansi lwesitho sangasese sowesifazane sangaphambili

Ucansi lwesitho sangasese sangemuva

Ucansi lomlomo

EX31A[i] [if EX3= female & EX28 = vaginal sex]

Ngesikhathi nigcina ukwenza ucansi lwesitho sangasese sangaphambili sowesifazane no [Partner #1], ngabe lokhu kwakunge, noma ngaphandle kwekhondomu?

Ngekhondomu

Ngaphandle kwekhondomu

EX31B[i] [if EX3= female & EX28 = anal sex]

Ngesikhathi nigcina ukwenza ucansi lwesitho sangasese sangemuva no[Partner #1], ngabe lokhu kwakunge, noma ngaphandle kwekhondomu

Ngekhondomu

Ngaphandle kwekhondomu

EX33[i] Uke u [Partner #1] wakutshela ngesimo sakhe seHIV?

Cha [JUMP TO E35[i]]
Yebo
Angazi

EX34[i] Sithini isimo sika [Partner #1] seHIV?

Une HIV
Akanayo iHIV
Angazi

SKIP TO E36[i]

EX35[i] Wawukholwa ukuthi isimo sika [Partner #1] seHIV sithini ngesikhathi nigcina ukwenza ucansi?

Ngicabanga ukuthi angaba nayo iHIV
Ngicabanga ukuthi akanayo iHIV
Angazi

EX36[i] Uke wena no [Partner #1] nakhaphana ukuyowenza loku okulandelayo [khetha okuyikho]

Ukuyolulekwa nokuhlola iHIV
Kuphoyinti lasemtholampilo wezocansi
Kuphoyinti lomtholampilo wokulapha iHIV
Umhlangano wezempilo wabantu besilisa abaya ocansini nabanye abantu besilisa
Umcimbi ohlelwe inhlangano yabantu besilisa abaya ocansini nabanye besilisa
Cha, akukho kulokhu okungenhla

Ukuqedela loluhlu sesizokubuza imibuzo emibalwa, emayelana nokwazana kwalabophathina oqeda kubabala

EX37 [IF >1 MALE] Ngokwazi kwakho, bakhona kulabantu besilisa abenza ucansi ndawonye, baphinde balwenze futhi nawe?

Yebo
Cha [SKIP TO NEXT SECTION]
Angazi [SKIP TO NEXT SECTION]

EX38 [IF >1 MALE REPORTED] Sicela uveze ukuthi ibaphi abenza ucansi ndawonye

<EX1> & <EX2> [IF BOTH MALE]
<EX1> & <EX3> [IF BOTH MALE]
<EX1> & <EX4> [IF BOTH MALE]
<EX2> & <EX3> [IF BOTH MALE]
<EX2> & <EX4> [IF BOTH MALE]
<EX3> & <EX4> [IF BOTH MALE]

NgesikhathiNgesikhathiNgesikhathiNgesikhathiNgesikhathingesikhathingesikhathikukangakiF. ULWAZI

NOKUZAZI KWAKHO NGE HIV NOKUPHEPHA KWEZOCANSI.

Loluhlu lwemibuzo elandelayo ingokwaziyo ngeHIV Kanye nokuthi ukuthola kulula kangakanani ukunakekela ezempilo zakho zocansi.

F1. Yonke lemibhalombiko IYIQINISO. <u>UBUKWAZI YINI LOKHU OKULANDELAYO?</u>

F1a. Ukulashwa okusebenzayo kwe HIV kwehlisa ukwendluliseka kwe HIV

Besengikwazi loku

Benginganasiciniseko sako

Bengingakakwazi loku

Angikuzwisisi loku

F1b. kuyenzeka ukuthi uthole I HIV ngecansi lwesitho sangansense sangemuva sabesilisa

Besengikwazi loku

Benginganasiciniseko sako

Bengingakakwazi loku

Angikuzwisisi loku

F1c. Kuyenzeka ukuthi uthole iHIV noma uyitop manenza ucansi lwesitho sangansense sangemuva sabantu besilisa.

Besengikwazi loku

Benginganasiciniseko sako

Bengingakakwazi loku

Angikuzwisisi loku

F2. Ingabe uvumelana kangakanani nalezitatamende ezilandelayo?

	Angivumelani nakho kakhulu	Nginokungavumelani nakho	Angivumelani futhi angiphikisi	Nginokuvuma	Ngivumelana nakho kakhulu
**F2a. 'Ucansi engilwenzayo luhlale luphephile ngendlela engifuna ngayo mina'**					
	○	○	○	○	○

**F2b. 'Ngingasenza isiciniseko sokuthi ngisebenzisa amacondom uma kumele asetshenziswe'**					
	◯	◯	◯	◯	◯
**F2c. 'ngesinye isikhathi ngiba nobunzima bokuthola ama condom mangiwadinga'**					
	◯	◯	◯	◯	◯
**F2d. 'ngesinye isikhathi ngiba nenkinga nama condom angangeni kahle'**					
	◯	◯	◯	◯	◯
**F2e. 'ngesinye isikhathi ngiba nenkinga ukuthola ilubhu yamanzi mangiyidinga'**					
	◯	◯	◯	◯	◯

G. EZEMPILO ZOCANSI NE HIV

Loluhlu lwemibuzo elandelayo ingokuhlolela iHIV

G1. Ukholwa ukuthi isimo sakho seHIV simephi namuhla?

Anginayo iHIV (ngicabanga ukuthi anginayo iHIV)

Nginayo iHIV (ngicabanga ukuthi nginayo iHIV)

Angicinisekanga

G2. Uke wahlola igazi uhlola iHIV?

Yebo

Cha [**jump to section G PART III**]

G3. Iyiphi inyanga nonyaka owagcina ukuhlolela iHIV?

[faka usuku MM/YYYY]

G4. Ukugcina kwakho ukuhlola iHIV, ugcinephi?

Esibhedlela noma eklinikhi yomphakathi

Esibhedlela noma eklinikhi ezimele

Endaweni yokuhlolela iHIV yomphakathi wonke

Endaweni yomphakathi lapho khona kuhlola khona abantu besilisa abathandana nabanye abantu besilisa.Endaweni lapho engihlangana nabangani (ebar noma eKlubhini)

Ngizihlole mina ekhaya

.

Okunye [Ngicela uchaze]**G5. Ukugcina kwakho ukuhlola iHIV, waneliseka kangakanani ngendlela ekumele kufihleke ngakhona kolwazi lokuhlolwa kwakho?**

Nganginelisekile kakhulu

Nganeliseka

Anginelisekanga

Anginelisekanga kakhulu

Angikhumbuli/ angiyicabanganga

G6. Ukugcina kwakho ukuhlola iHIV, waneliseka kangakanani ngabasebezi bomtholampilo ngenhlonipho ababenayo?

Nganginelisekile kakhulu

Nganeliseka

Anginelisekanga

Anginelisekanga kakhulu

Angikhumbuli/ angiyicabanganga

G8. Yayithini imiphumela yokuhlola yamaduzane yeHIV?

Anginayo IHIV
Nginayo iHIV
Angazi

Section G Part i: About being HIV positive

Loluhlu lwemibuzo elandelayo imayelana nokuzithola uneHIV

G10. Kwakungayiphi inyanga noma unyaka uma uthola ukuthi unayo I IHV?
[faka ususku MM/YY]

G11. Kusukela ngalesikhathi uthola ukuthi unayo iHIV, usuke wadluliselwa yini emtholampilo ukuyohlolisisa ngayo iHIV noma ngesimo sakho sempilo? Ngokuthi 'Emtholampilo' sisho icliniki lapho ubona khona udokotela noma osebenza khona ngokuzinakekela ngokuzilapha.

Yebo
Cha

G12. Kusukela ngesikhathi waqala ukuthola ukuthi une HIV, uke wavakashela umtholampilo nge HIV noma izifo ezihambelanayo??

Yebo **[JUMP TO G14]**
Cha

G13 Kungani ungavakashelanga umtholampilo emva kokuthi uthunyelwe khona?? (khetha okungenayo)

Indlela yokuziphatha yabantu abasebenza emtholampilo kuma MSM
Indlela yokuziphatha yabasebenzi basemtholampilo kubantu abaphila neHIV
ukwesaba ukubonwa mangiya emtholampilo
Indlela ende ukuya emtholampilo
Ukudula komtholampilo/ukuhlola
Ukudula ukuya emtholampilo
Ukudula komuthi
Ukusaba ukugula okuza nokuthatha umuthi
Ukusaba ukuthi umuthi uzobonwa abantu
Angikholwanga ukuthi umuthi uyasebenza
Angikholwanga ukuthi ngiyawudinga umuthi
Ezinye izizathu [sicela ucacise________________]

G14. Kusukela ngelanga owazi ngalo ukuthi unayo iHIV, kwaba duzane kangakanani ukubonana nomuntu osebenza emtholampilo ngokunakekela iHIV?

Ngalelo langa engazingalo ngesimo sami
Emavikini amabili emuva kokwazi ngesimo sami
Emaviki ayi2 kuya ku4 emuva kokwazi ngesimo sami
Izinyanga eziwu1 kuya ku3 emuva kokwazi ngesimo sami
Ezinyangeni eziwu 3 kuya ku 12 emuva kokwazi ngesimo sami

Ngaphezulu konyaka ngazi ngesimo sami

ADDED Angikaze ngibonane nomuntu osebenza emtholampilo ngokunakekela iHIV

G15. Uye kuphi mawuqala ukuvakashela umtholampilo ngalenhloso?

Esbhedlela somphakathi noma iKliniki

Esbhedlela esizimele noma ikliniki

Ekliniki yabesilisa abaya ocansini nabanye besilisa

UKUNAKEKELA IHIV OKUQHUBEKAYO

G16. Ugcine nini ukubonana nowezempilo osemthethweni mayelana nokunakekelwa kwe HIV?

Kulezinyanga eziwu 6 zokugcina

Phakathi kwezinyanga eziwu 6 kuya kweziwu 12

Phakathi kweminyaka eyi1 kua ku 2

Eminyakeni endlula eyiwu 2 eyedlule

G17. Uye kuphi ngesikhathi ugcina emtholampilo ngalenhloso?

Esbhedlela somphakathi noma iKliniki

Esbhedlela esizimele noma ikliniki

Ekliniki yabesilisa abaya ocansini nabanye besilisa

G18. Ngesikhathi ugcina ukuya emtholampilo ukuyohlola ngokunakekelwa kwe HIV, waneliseka ngobumfihlo bamasevisi abo?

Nganeliseka kakhulu

Nganeliseka

Anginelisekanga

Anginelisekanga kakhulu

Angikhumbuli/ Angicabangi ngakho

G20. Ngesikhathi ugcina ukuvakashela umtholampilo ngokunakekela iHIV, waneliseka yini ngenhlonipho abasebenzi abakubonisa yona?

Nganeliseka kakhulu

Nganeliseka

Anginelisekanga

Anginelisekanga kakhulu

Angikhumbuli/ Angicabangi

G22. Usake wawahlola ukuthi ahamba kanjani amasosha emzimbeni. Lohlolo lubizwa nge CD4 test?

Yebo

Cha

Angazi kahle

G22a. [If G22 = yes] Ugcine nini ukwenza lolo hlolo lwe CD4?

Kulezinyanga eziyisithupha ezedlule

Phakathi kwenyanga eziyisithupha kuya ezinyangeni eziyishumi nambili ezedlule

Phakathi konyaka noma iminyaka emibili eyedlule
Ngaphezu kweminyaka emibili endlule

G23. [If G22 = yes]Sasithini isibalo sakho se CD4 ngesikhathi uyohlola?
Ngaphezu kuka 500
Phakathi kuka 350 kuya ku 500
Ngaphansi kuka 350
Bangitshela kodwa angisakhumbuli
Bangitshela kodwa angizwisisanga kahle
Angitshelwanga imiphumela

G24. Usake wahlolelwa, inani lokutheleleka ngeHIV egazini. Loku kubizwa nge viral load.
Yebo
Cha
Angazi kahle

G24a. [If G24 = yes] Ugcine nini ukuthola imiphumela yakho ye viral load?
Angizange
Ezinyangeni eziwu 6
Phakathi kwezinyanga eziwu6 kuya ku 12
Phakathi konyaka neminyaka ewu 2
Sekudlule iminyaka ewu 2

G25. [If G24 = yes] Ibithini imiphumela yokuhlolwa kwe viral load mawugcina ukuhlola?
Ayibonakalanga
Yabonakala
Bangithsela kodwa angikhumbuli
Bangitshela kodwa angizwisisanga
Abangitshelanga imiphumela

UKKWELASHWA KWE HIV (ART)
Loluhlu lwemibuzo elandelayo ingokuthatha imithi edambisa iHIV (ART, HAART).
G26. Ingabe sewuqalile yini ukuthatha ama antiretroviral (ngesinye isikhathi aziwa ngokuthi yi ART [antiretroviral treatment] noma HAART [highly-active antiretroviral therapy] eHIV?
Yebo
Cha **[JUMP TO G28]**

G27. Uthe uzwa ngesimo sakho seHIV mhlaka <G8 MM YY>. Waqala duzane kanganani ukuthatha amaART akho emuva kwalokho?
Ngalelo langa engazingalo ngesimo sami
Emavikini amabili emuva kokwazi ngesimo sami
Emavikini awi2 kuya ku4 emuva kokwazi ngesimo sami
Izinyanga eziwu1 kuya ku3 emuva kokwazi ngesimo sami
Ezinyangeni eziwu 3 kuya ku 12 emuva kokwazi ngesimo sami

Ngaphezulu konyaka ngazi ngesimo sami

[JUMP TO SECTION G PART IV]

G28. Ingabe okusizayo ngendaba zempilo usekululekile ukuthi uqale uthathe ama antiretroviral treatment (ART)?

Yebo

Cha [JUMP TO SECTION G PART IV]

G29. Ingabe yini eyakwenza ukuthi ungaqali ukuthatha umshanguzo wama antiretroviral treatment (ART)? [Khetha konke okungenayo]

Indlela yokuziphatha yabantu abasebenza emtholampilo kuma MSM

Indlela yokuziphatha yabasebenzi basemtholampilo kubantu abaphila neHIV

Ukwesaba ukubonwa mangiya emtholampilo

Indlela ende ukuya emtholampilo

Ukudula komtholampilo/ukuhlola

Ukudula ukuya emtholampilo

Ukudula komuthi

Ukusaba ukugula okuza nokuthatha umuthi

Ukusaba ukuthi umuthi uzobonwa abantu

Angikholwanga ukuthi umuthi uyasebenza

Angikholwanga ukuthi ngiyawudinga umuthi

Ezinye izizathu [sicela ucacise________________]

[JUMP TO SECTION G PART IV]

G30. Ingabe manje uyayithatha iantiretroviral treatment (ART)?

Yebo

Cha

[If G30 = no]Uyeke nini ukuthatha ama antiretroviral treatment (ART)?

Kulezinyanga eziyisithupha ezedlule

Phakathi kwenyanga eziyisithupha kuya ezinyangeni eziyishumi nambili ezedlule

Phakathi konyaka noma iminyaka emibili eyedlule

Ngaphezu kweminyaka emibili endlule

[If G30 = no]

G32. Ingabe yini eyakwenza ukuthi uyekele ukuthatha iantiretroviral treatment? [Khetha okungenayo]

Indlela yokuziphatha yabantu abasebenza emtholampilo ebhekiswe kuma MSM

Indlela yokuziphatha yabasebenzi basemtholampilo kubantu abaphila neHIV

Ukwesaba ukubonwa mangiya emtholampilo

Indlela ende ukuya emtholampilo

Ukudula komtholampilo/ukuhlola

Ukudula ukuya emtholampilo

Ukudula komuthi

Ukusaba ukugula okuza nokuthatha umuthi
Ukusaba ukuthi umuthi uzobonwa abantu
Angikholwanga ukuthi umuthi uyasebenza
Angikholwanga ukuthi ngiyawudinga umuthi
Ezinye izizathu [sicela ucacise________________]

[If G30 = yes] G31. Iningi leziguli likuthola kunzima ukuthatha imithi yeHIV belandela indlela abatshelwe ngayo. Ngabe mangaki amadozi yemithi yakho yeHIV ongawathathanga kulezizinsuku eziwu7 ezedlule?

[Fa]ka inani lama dozi

ISIGABA G INXENYE II. NGOKUNGABI NE HIV

Loluhlu lwemibuzo elandelayo imayelana nokungabi nayo iHIV

G33. Kullezinyanga eziwu12 ezedlule, uye kangakhi ukuyohlola iHIV?

[faka inani]

G34. Mangabe uyanquma ukuhlolela iHIV futhi, ungafuna ukuhlolelaphi?

Esbhedlela somphakathi noma iKliniki
Esbhedlela esizimele noma ikliniki
Isevisi yomphakathi yokuhlola iHIV
Isevisi yomphakathi yokuhlola iHIV yabesilisa abaya ocansini nabesilisa
Endaweni lapho ngihlangana nabangani (e.g. bars or clubs)
Ekhaya

G35. Uma unganquma ukuhlola iHIV futhi, ubani ongathanda ukuthi akwenze lohlolo?

Udokotela noma umsebenzi wezempilo
Unesi
Ikhansela
Osebenzela umphakathi wama MSM
Imina [i.e. ukuzihlola]

[JUMP TO SECTION G PART IV]

ISIGABA G INXENYE III: NGOKUNGAYIHLOLELI NHLOBO IHIV

Loluhlu lwemibuzo lungokungayihloleli nhlobo iHIV

G36. Kungani ungakahloli isimo sakho se HIV??

[enter text]
Ngikhathazekile ukuthi angeke bangiphathe ngenhlonipho emtholampilo
Akubalulekike kimi ukuthi ngazi
Ngoba ngicabanga ukuthi isimo sami siyafana neskaphathini wami

Angazi ukuthi ngiyosihlola kuphi
Ngiyasaba ngoba ngicabanga ukuthi nginayo iHIV
Ngiysaba ukuthi abantu ngeke basangiphatha kahle uma ngiyohlola
Ngisaba ukuthi uma ngineHIV abantu ngeke basangiphatha ngendlela ejwayelekile
Kunganibangela izinkinga ebudlelwaneni bami
Anginaso isizathu esingangenza ngibe neHIV
Esinye isizathu (isho________________)

G37. Uzethemba kangakanani ukuthi ungayihlolela iHIV makungenzeka ufune?
Ngizethemba kakhulu
Ngiyazethemba
Ngizethemba kancane
Angizethembi nhlobo
Angazi

G38. Uma ungancuma ukuthi uyozihlola ngelinye ilanga, ungathanda ukuhlolaphi?
Esbhedlela somphakathi noma iKliniki
Esbhedlela esizimele noma ikliniki
Ekliniki yabesilisa abaya ocansini nabanye besilisa
Isevisi yomphakathi yokuhlola iHIV yabesilisa abaya ocansini nabesilisa
Endaweni lapho ngihlangana nabangani (e.g. bars or clubs)
Ekhaya

G39. Uma ungancuma ukuthi uyohlola ngelinye ilanga, ungathanda ukuhlolwa ngubani?
Udokotela noma umsebenzi wezempilo
Unesi
Ikhansela
Osebenzela umphakathi wama MSM
Mima [i.e. ukuzihlola]

SECTION G PART IV: EZINYE IZIFO EZITHATHELELANA NGOCANSI

G40. Kulezinyanga eziwu 12 ezedlule, uke waphuma ubovu esithweni sakho somzimba sangansense sangaphambili noma kube buhlungu uma uchama?
Yebo
Cha **[JUMP to G35]**

G41. . Ingabe unazo lezimpawu namuhla
Yebo
Cha

G42. Kulezinyanga eziyishumi nambili ezedlule, ingabe uke waphuma ubovu noma igazi esithweni sakho somzimba sangansense sangemuva noma uzwe ubuhlungu obungabekezeleleki uma wenza ucansi esithweni sakho somzimba sangansense sangemuva?

Yebo
Cha **[JUMP to G44]**

G43. Ingabe unazo lezimpawu namuhla?

Yebo
Cha

G44. Kulezinyanga eziyishumi nambili ezedlule, uke wabona izilonda esithweni sakho somzimba sangasese sangaphambili nesesithweni sakho somzimba sangasese sangemuva?

Yebo
cha

[IF A16 = A17 JUMP TO NEXT SECTION]

SECTION G PART V: TRANSGENDER SEXUAL HEALTH ACCESS

G45. Uzethemba kanjani ukuthi ungathola ukukhanselwa ngokobulili bakho?

Ngizethemba kakhulu
Ngiyazethemba
Ngizethemba kancane
Angizethembi nhlobo
Angazi

G46. Ingabe njengamanje uyawasebenzisa amahomoni noma amahomoni avimba ukwelapha?

Yebo
Cha **[SKIP TO G48]**

G47. Ingabe ukutholaphi lokwelapha?

Isibhedlela somphakathi
Isibhedlela sangansese
Ngawathenga ekhemisi
Ngawathenga kuinthanethi
Ngawathola kubangani

[SKIP TO G49]

G48. Uzethemba kangakanani ukuthi ungawathola amahomoni noma ukwelashwa okuvimba amahomoni e<IZWE> uma ubuwafuna?

Ngizethemba kakhulu
Ngiyazethemba
Ngizethemba kancane
Angizethembi nhlobo
Angazi

G49. Usuke wahlinzwa ukuze ushintshe ubulili?

Yebo
Cha **[SKIP TO G51]**

G50. Where were you able to access these services?
Esbhedlela somphakathi khona kulelizwe
Isibhedlela esizimele kulelizwe
Esbhedlela kwelinye izwe
[JUMP TO NEXT SECTION]

G51. Uzethemba kangakanani ukuthi ungakwazi ukuthola ukuhlinzwa okuhlobene nobulili bakho?
Ngizethemba kakhulu
Ngiyazethemba
Ngizethemba kancane
Angizethembi nhlobo
Angazi

H. Post Exposure Prophylaxis (PEP) and Pre Exposure Prophylaxis (PrEP)

Loluhlu lwemibuzo elandelayo inge Post Exposure Prophylaxis-ebuye yaziwe nge PEP.

H1. Lesitatimenti esilandelayo siyiqiniso. Ingabe ubusukwazi lokho?

I Post Prophylaxis (PEP) amaphilisi athathwa inyanga yonke avikela ukuthi umuntu angatheleleki ngeHIV uma bevulelekile kuyona, (kufana nokuya ecansini ungafakanga icondom). iPEP ifuna ukuthathwa emuva kokuvuleleka engcupheni ye HIV.

Bengivele ngikwazi lokhu
Bengingaso isiciniseko salokhu
Bengivele ngingakwazi lokhu
Angikuzwisisi lokhu

H2. Usuke wazama ukuthola iPEP?

Yebo
Cha
Angazi

H3. [If yes to H2] Usuke wayithatha iPEP phambilini?

Yebo
Cha
Angazi

H4. [If yes to H3] IPEP uyithathe amalanga ayingakhi? (Uma uyithathe kaningi kunakodwa, cabanga ngeseikhathi owagcina ngayo ukuyithatha)

[faka inani]

H5. [IF G1 = NEGATIVE or NOT SURE] Uma ucabanga ukuthi uvezekile ku HIV ungazi ukuthi uzoyitholaphi I PEP?

Yebo
Cha
Angazi

H. Pre Exposure Prophylaxis (PrEP)

The next set of questions is about Pre Exposure Prophylaxis - which is also known as PrEP.

H6. Lesitatamende esilandelayo siyiqiniso . Ubusuwazi ngaloku?

. iPre exposure prophylaxis (PrEP) ifaka phakathi umuntu onganayo iHIV othatha ipilisi ngesikhathi esihambayo ukuzivikela ekutholeni iHIV. Abantu abaningi abasebenzisa iPrEP onke amalanga. IPrEP idinga ukuthi uyithathe ngaphambi kokuya ocansini ukuze ikwazi ukusebenza.

Besengikwazi lokhu
Benginganaso isiciniseko salokhu
Bengingakakwazi lokhu
Angikuzwisisi lokhu

PrEP ihlukile kune PEP, iPEP ithathwa emuva kokuvuleleka iPrEP yona ithathwa ngaphambi kokuvuleleka.

H7. Uke wazama ukuthola iPrEP?

Yebo
Cha **[JUMP TO H10]**
Angazi **[JUMP TO H10]**

H8. Usake wanikezwa iPrEP?

Yebo
Cha
Angazi

H8. Usake wayithatha iPrEP?

Yebo, futhi ngisayisebenzisa
Yebo, ngiyekile ukuyisebenzisa
Cha **[JUMP TO H10]**
Angazi **[JUMP TO H10]**

H9. Wayitholaphi iPrEP?

Udokotela esibhedlela somphakathi noma ekliniki
Udokotela esibhedlela esizimele noma ekliniki
Enhlanganweni yomphakathi
Kuwebhusayithi ezimisele (shano ukuthi yiphi)
Kwenye indawo (shano ukuthi kuphi)

[JUMP TO NEXT SECTION]

H10. [IF G1 = NEGATIVE or NOT SURE] Uma iPrEP ingenziwa ukuthi itholakale ucabanga ukuthi ungayisebenzisa yini?

Cishe kakhulu
Cishe nje
Anginasiciniseko
Cishe Kancane
Angiboni nhlobo

H11. Uma iPrEP ingenziwa ukuthi itholakale, ungathanda ukuyitholaphi?

Udokotela esibhedlela somphakathi noma ekliniki
Udokotela esibhedlela esizimele noma ekliniki
Enhlanganweni yomphakathi
Ekhemisi
Kuwebhusayithi ezimisele (shano ukuthi yiphi)
Kwenye indawo (shano ukuthi kuphi)

ISAHLUKO I. UKUSETSHENZISWA KOTSHWALA

I1. Uziphuza kangakanani izinto ezinotshwana

Angikaze

Njalo ngenyanga

Kabili ukuya kane enyangeni

Kabili ukuya kathathu ngesonto

Kane noma kaningi ngesonto

I2. Uma uphuza utshwala, uphuza iziphuzo ezingakhi?

1 noma 2

3 noma 4

5 noma 6

7 kuya ku 9

10 noma adlulayo

I3. Kukangaki lapho uphuza khona iziphuzo eziba wu6 noma ezindlulayo ngesikhathi esisodwa?

Angikwenzi

Kuyandlula inyanga

Ngenyanga

Ngesonto

Ngelanga noma cishe onke amalanga

I4. Kukangaki kulonyaka odlule lapho uzithole khona ungakhoni ukuyeka ukuphuza uma sowucalile?

Angikwenzi

Kuyandlula inyanga

Ngenyanga

Ngesonto

Ngelanga noma cishe onke amalanga

I5. Kukangaki kulonyaka odlule lapho uzithole khona uhluleka ukuziphatha ngendlela ejwayelekile ngoba ubuphuzile?

Angikwenzi

Kuyandlula inyanga

Ngenyanga

Ngesonto

Ngelanga noma cishe onke amalanga

I6. Kukangaki kulonyaka odlule lapho khona ubudinga isiphuzo esisodwa ukuze ukwazi ukuba nguwe emuva kokuphuza kakhulu?

Angikwenzi

Kuyandlula inyanga

Ngenyanga

Ngesonto

Ngelanga noma cishe onke amalanga

I7 Kukangaki kulonyaka odlule lapho khona udliwe khona isazela noma ukuzisola emuva kokuphuza?

Angikwenzi

Kuyandlula inyanga

Ngenyanga

Ngesonto

Ngelanga noma cishe onke amalanga

I8. Kukangaki kulonyaka odlule lapho ungakwazanga ukukhumbula izinto ezenzeke ngayizolo ngenxa yokuthi bewuphuzile?

Angikwenzi

Kuyandlula inyanga

Ngenyanga

Ngesonto

Ngelanga noma cishe onke amalanga

I9. Uke noma omunye umuntu walimala ngoba wena ubuphuzile?

Cha

Yebo, kodwa hhayi kulonyaka odlule

Yebo, kulonyaka odlule

I10. Ngabe isihlobo, umngani, udokotela noma isisebenzi sezempilo bake babonisa ukukhathazeka ngokuphuza kwakho?

Cha

Yebo, kodwa hhayi kulonyaka odlule

Yebo, kulonyaka odlule

ISAHLUKO J. UKUSEBENZISA KWEZIDAKAMIZWA

Loluhlu lwemibuzo elandelayo ingokusebenzisa kwakho ugwayi noma ezinye izidakamizwa.

J1. Ngicela ucacise uma usake wakusebenzisa lokhu okulandelayo:

Isidakamizwa	Angikaze	Kulenyanga edlule	Phakathi konyaka odlule kodwa hhayi kulenyanga edlule	Kudlule cishe unyaka
Tobacco	◯	◯	◯	◯
Cannabis (grass, weed, herb, ndom, bhang, ganja, dagga, zol, insangu)	◯	◯	◯	◯
Khat (miraa, veve, mogoka)	◯	◯	◯	◯
Ecstasy (E, umgwinyo, happy pill, disco biscuit, Adam)	◯	◯	◯	◯
Amphetamine (speed, gavana)	◯	◯	◯	◯
Crystal methamphetamine (crystal, ice, tina, meth, taptap, crank)	◯	◯	◯	◯
Heroin (smack, mud, brown sugar)	◯	◯	◯	◯
Mephedrone (meow meow, plant food, bubbles, kitty cat)	◯	◯	◯	◯
GHB/GBL (G, liquid ecstasy, soap)	◯	◯	◯	◯
Cocaine or Crack cocaine (rock)	◯	◯	◯	◯
Rohypnol (mchele, roofies, forget pill)	◯	◯	◯	◯
Poppers (liquid gold)	◯	◯	◯	◯
Benzene	◯	◯	◯	◯

K. UKUPHILA KWENGCONDO

Kulamaviki amabili okugcina uke wahlushwa okunye kwaloku okulandelayo:

	Abfikwenzi Nhlobo	Amalanga ambalwa	Ngaphezu kwehhafu yalamalanga	Cishe onke amalanga
**K1. ngimomdlandla omncane ekwenzeni izinto**				
	○	○	○	○
**K2. ngizizwa ngiphansi ngikhathazekile futhi nginganathemba**				
	○	○	○	○
**K3. Inkinga yokulala noma ukuhlala ngilele, noma ukulala ngokweqile**				
	○	○	○	○
**K4. ukuzizwa ngikhathele noma ngimanandla amancane**				
	○	○	○	○
**K5. Ukungadleki noma ukudla kakhulu**				
	○	○	○	○
**K6. ngizizwa kabi ngami, noma ngizizwe njengesehluleki, noma ngizicekele phansi ngacekela nomndeni wami phansi**				
	○	○	○	○
**K7. Inkinga yokugxilisa ingqondo ezintweni ezifana nokufunda iphephandaba noma ukubheka umabonakude**				
	○	○	○	○
**K8. Ukuhamba noma ukukhuluma kancane ukuthi abantu banganaki nokunaka? Noma okuhlukile, okuba ukungahlaliseki nokungaphumuli okufaka nokuhambahamba ukudlula injwayelo.**				
	○	○	○	○
**K9. Imicabango yokuthi kungabangcono mawungafa noma yokuzilimaza ngendlela thize.**				
	○	○	○	○

K10. Uma kukhona inking oyikhethile, ubunzima obunganani lezinkinga ezibenzile ekwenzeni umsebenzi wakho, ukunakekela izinto ekhaya, noma ukuzwana nabantu?

Akunzima Nhlobo
Kunzima nje
Kunzima kakhulu
Kunzima ngokweqile

L. UKUQAGELA INANI LABANTU

Manje sifisa ukukubuza ukuthi uke wasebenzisa amasevisi akhethekile kulamalanga. Izimpendulo zalemibuzo zisosinceda ukuthi sithole inani labantu abesilisa abaya ocansini nabanye abantu besilisa. Ayikho imininingwane engawe ezodluliswa noma ezocelwa kulamasevisi ukucagela lenani.

[SHOW IF SITE = NAIROBI]

[SHOW IF SITE = NAIROBI]

L1. Mhla ziwu [reference date], wabhalisa njengelunga eqenjini lefacebook le 'ISHTAR-MSM'?

- Yebo
- Cha
- Angazi

L2. Uke waba nephoyinti e [Liverpool VCT or ISHTAR clinic] Phakathi kuka [enter reference period]

- Yebo
- Cha
- Angazi

[SHOW IF SITE = SOUTH AFRICA]

L1. Ngomhlaka [reference date], Ingabe wawubhalisele ukuba yinxenye yeqembu lefacebook i'Black Men Bold and the Beautiful'?

- Yebo
- Cha
- Angazi

L2. Ngomhlaka [reference date], wabhalisa ukuba ingxenye yeqembu lefacebook i'Johannesburg Gays' ?

- Yebo
- Cha
- Angazi

L3. Ngomhlaka [reference date], wabhalisa ukuba ingxenye yeqembu lefacebook i'Soweto Gays'?

- Yebo
- Cha
- Angazi

L4. Ngomhlaka [reference date], wawulandela i'We the Brave' ku Facebook?

- **Yebo**
- **Cha**
- **Angazi**

L5. Ngomhlaka [reference date], wawulandela i'Health 4 Men' ku Facebook?

- **Yebo**
- **Cha**
- **Angazi**

L6.Uke wavakashela umtholampilo we ANOVA Health 4 Men ngaphakathi kuka[enter reference period]

Yebo
Cha
Angazi

[If L6 = yes] L7. Iyiphi iklinikhi yeANOVA Health 4Men owayivakashela?

[Amaklinikhi akhona]
Zola
Chiawelo
Yeoville
Other__________

M. UKUJABULA KWEZOCANSI

M1. Ujabule kangakanani ngimpilo yakho yezocansi manje?

Ngijabule kakhulu

Ngijabulile nje

Anginasiciniseko/angazi

Angijabulile nje

Angijabulile kakhulu

M2. Yini into eyodwa engakusiza ukuthi impilo yakho yezocansi ibe ngcono?

[Bhala]

M3. Iyiphi indoda ebukeka kahle kunawo wonke emhlabeni?

[Bhala]

Siyabonga kakhulu ngokubamba kwakho iqhaza kulolucwaningo. Uma uzwa ngathi knona amaphutha owenzile ufisa ukubuyela emuva uyowalungisa, ngicela uwasho kumcwaningi ozobe ekusiza. Uma loqwaningo lukwenza ucabange ngemibuzo engempilo yakho nangalocwaningo, sicela ubuze umcwaningi.

Luyaphela lapho uhla lemibuzo.

TRANSFORM Survey Instrument: IMOJULA YOKUPHA AMAKHUPHONI

Ilanga Lokuhlolwa: [unyaka/inyanga/usuku]

Igama lomhloli: ___________________

Inombolo yepasi kambambiqhaza: ______________________

kulolucwaningokulolucwaningolelikhuphoni

Ukuvakasha Kokubuya

B1. Mangaki amanye amadoda aya ocansini namanye owaziyo okewaxoxisana nawo kulenyanga edlule? Ngokuthi 'owaziyo', sisho ukuthi umuntu owazi igama lakho futhi nawe wazi elakhe, futhi ngokuthi 'uxoxisane naye' sisho ukuthi omazi ngesicu noma enikhuluma naye ocingweni, ukuthumelana imilayezo noma elayinini.

[faka inimbolo]

B2. Mangaki [B1] kulamadoda oke wahlangana nawo ngokwesiqu?

[faka inombolo]

B3. Mangaki [B2] kulamadoda aneminyaka ewu18 ukuya phezulu?

[faka inombolo]

Q9. Mangaki [B3] kulamadoda ahlala e [Johannesburg/Nairobii]?

[faka inombolo]

Q10. Mangaki [B4] kulamadoda oke wawabona kulamaviki amabili adlule?

[faka inombolo]

UKUDLULISA AMAKHUPHONI (ABANQABILE)

Sifuna ukukubuza ngabantu abangavumanga ukwamukela ikhuphoni kuwe.

Q11 Mangaki amadoda obufuna ukuwapha lelikhuphoni anqabile ukuyithatha?

[faka inombolo]

Q12 For each of [Q11] Abayithathanga ngani lelikhuphoni?

Ucabanga umuntu wesilisa wokuqala owazama ukumunika ikhuphoni wanqaba ukulithatha,

Yini engalithathanga ikhuphoni kuwe? [khetha konke okungenayo]

Uthe uselibambile iqhaza kulolucwaningo

Uthe useyitholile lelikhuphoni komunye umuntu

Uthe akayona indoda eya ocansini namanye amadoda

Uthe uneminyaka engaphansi kwa 18
Uthe akahlali edolobheni engihlala kulo
Akanaso isifiso sokubamba iqhaza kulolucwaningo
Uthe akanayo imali eyanele ukuthi angabamba iqhaza
Esinye isizathu-sisho
Akukho kulokhu nokungenhla

UKUDLULISA AMAKHUPHONI (ABAWAMKELILE)

Manje sicela ukukubuza ngabantu abalithathile lelikhuphoni kuwe.

Q13. Bangaki abesilisa obaphe lelikhuphoni abalithethe?

[faka inombolo 0-3]

IF Q13>0 Lomuntu omuphe ikhuphoni yokuqala

Q14i: Ucabanga ukuthi lomuntu ubezokunika ikhuphoni kube ubebambe iqhaza kuqala kunawe kulolucwaningo?

Yebo
Cha

Q14i: Sisacabanga ngalomuntu wokuqala omunikeze ikhuphoni walamukela:

Lomuntu ngabe uneminyaka engaphezu kwewu 30 na?

Yebo
Cha
Angazi

Q14i: Sisacabanga ngalomuntu wokuqala omunikeze ikhuphoni walamukela:
Yini ekwenze wanquma ukupha lomuntu ikhuphoni? Khetha isizathu esibaluleke kakhulu

Bekawokuqala okhonayo ukungena kulolucwaningo
Ungumngani wami osondele kakhulu
Unguphathina wami
Ubedinga lemali yokubamba iqhaza
Bengicabanga ukuthi khona azokuzuza kulolucwaningo
Ngimucele ngokuvulekile yena weza kimina
Okunye(sicela usho isizathu__________________)

LOOP Q14i KWABANYE OSAZOBAPHA AMAKHUPHONI

[IF Q14>1] Okufaka lomuntu wesibili ozomupha ikhuphoni

[IF Q14>2] Okufaka umuntu wesithathu ozomupha ikhuphoni
